# The metabolism and role of free fatty acids in key physiological processes in insects of medical, veterinary and forensic importance

**DOI:** 10.7717/peerj.12563

**Published:** 2021-12-22

**Authors:** Agata Kaczmarek, Mieczysława Boguś

**Affiliations:** 1 Witold Stefański Institute of Parasitology, Polish Academy of Sciences, Warsaw, Poland; 2 Biomibo, Warsaw, Poland

**Keywords:** Adaptation to cold, Fatty acid transport protein, Free fatty acid, Insect physiology, Lipophorin, Polyunsaturated fatty acid

## Abstract

Insects are the most widespread group of organisms and more than one million species have been described. These animals have significant ecological functions, for example they are pollinators of many types of plants. However, they also have direct influence on human life in different manners. They have high medical and veterinary significance, stemming from their role as vectors of disease and infection of wounds and necrotic tissue; they are also plant pests, parasitoids and predators whose activities can influence agriculture. In addition, their use in medical treatments, such as maggot therapy of gangrene and wounds, has grown considerably. They also have many uses in forensic science to determine the minimum post-mortem interval and provide valuable information about the movement of the body, cause of the death, drug use, or poisoning. It has also been proposed that they may be used as model organisms to replace mammal systems in research. The present review describes the role of free fatty acids (FFAs) in key physiological processes in insects. By focusing on insects of medical, veterinary significance, we have limited our description of the physiological processes to those most important from the point of view of insect control; the study examines their effects on insect reproduction and resistance to the adverse effects of abiotic (low temperature) and biotic (pathogens) factors.

## Introduction

Due to varied feeding habits of insects, especially in the larval stage (sarcophagy, coprophagy, necrophagy), insects have considerable veterinary and medical impact as obligatory and facultative parasitoids, predators and myiasis-causing factors (Kamut & Jezierski, 2014; Scholl, Colwell & Cepeda-Palacios, 2018; Silveira et al., 2019). Several species, like mosquitoes, cockroaches or flies, are synanthropic and may be responsible for the mechanical transmission of pathogens to food and the human body (Higgs & Vanlandingham, 2016; Scholl, Colwell & Cepeda-Palacios, 2018; Gerhardt & Hribar, 2019; Huang, Higgs & Vanlandingham, 2019; Donkor, 2020). Other species are hematophagous and can disseminate vector-borne diseases, like malaria, Dengue or Chagas’ Disease (Majerowicz & Gondim, 2013).

In addition to their epidemiological importance, the flies from the Families Calliphoridae (blow flies), Sacrophagidae (flesh flies) and Muscidae (house flies) are also convenient tools for forensic studies, and can be used for detecting drugs or other toxic substances (entomotoxicology) (Byrd & Sutton, 2020; Hodecek, 2020), while the larvae of other species, such as *Lucilia sericata* (Diptera: Calliphoridae) or *Protophormia terraenovae* (Diptera: Calliphoridae), are also used as maggot therapy in the treatment of gangrene and wounds (Stadler, 2020; Zubir, Holloway & Noor, 2020). Some insects, mostly *Drosophila melanogaster* (Diptera: Drosophilidae) but also *Galleria mellonella* (Lepidoptera: Pyralidae), *Apis mellifera* (Hymenoptera: Apidae) and *Bombyx mori (Lepidoptera: Bombycidae)*, are considered as model organisms in medical and veterinary research (Menzel, 2012; Abdelli, Peng & Keping, 2018; Nainu et al., 2020; Wojda et al., 2020; Younes et al., 2020; Beer & Helfrich-Forster, 2020).

Due to their negative impact on human and livestock health, there is a need to better understand the physiological processes governing their development and reproduction.

FFAs are a diverse group of lipids which play important roles in maintaining homeostasis and managing cellular processes. By restricting our scope to insects of medical, veterinary and forensic importance, the present article focuses on the key challenges faced by human populations exposed to these species and potential control methods. The ability of insects to adapt to cold conditions is key to their colonisation success, and the role of FFAs in these mechanisms should be highlighted. In addition, any potential disruption of this process might be employed to control the insect population. In addition, most of insects with medical, veterinary and forensic significance live in environments with high numbers of pathogenic microorganisms, such as decaying animal tissues or faeces, and previous studies have highlighted that insect cuticular FFAs significantly influence survival. As any manipulation of FFA synthesis or transport may be used to significantly decrease the insect population, any discussion of insect resistance should include data the role of FFAs. Moreover, a more thorough understanding of the role of FFAs in insect resistance and reproduction could serve as an effective tool for controlling insect-borne pathogens.

The presence and level of FFAs demonstrate considerable variation between insect developmental stages, even in the same species (Soulages & Wells, 1994; Gołębiowski et al., 2007; Gołębiowski et al., 2008; Gołębiowski et al., 2010; Gołębiowski et al., 2012b; Gołębiowski et al., 2013b; Gołębiowski et al., 2013a; Gołębiowski et al., 2015; Gołębiowski et al., 2016; Urbanek et al., 2012; Gutierrez et al., 2015; Paszkiewicz et al., 2016b; Sönmez, Güvenç & Gülel, 2016; Wrońska et al., 2018; Wojciechowska, Stepnowski & Gołębiowski, 2019; Cerkowniak et al., 2020; Giannetto et al., 2020; Kaczmarek et al., 2020a; Wojciechowska & Gołębiowski, 2020). Internal fatty acids are essential components of cell and organelle membranes; they are known to act as energy sources, and precursors for various waxes and pheromones, secondary metabolites and even defensive secretions (Blomquist & Bagnères, 2010; Ginzel et al., 2021).

The FFAs found in the cuticle are a diverse set of lipids whose content and composition vary considerably according to diet and climate. They perform a range of essential functions associated with well-being of insects, particularly minimizing transpiration and protecting terrestrial insects from desiccation (Gibbs & Rajpurohit, 2010). Cuticular lipids also play important roles in biochemical, physiological and semiochemical processes. They are also used as cues for recognising species, nest mates and castes. In addition, they are considered as a precursor of pheromones needed for sexual attraction and epideictic activity, such as display behaviour, as well as territorial markers, alarm signals and defensive chemicals. They are also believed to support thermoregulation, predator–prey and parasitoid-host interactions, and to enable insect camouflage and mimicry (Blomquist & Bagnères, 2010). Most importantly for the purposes of this paper, the lipid profile of the insect cuticle can also determine the effectiveness of fungal invasion (Kerwin, 1982; Gołębiowski et al., 2020; Gołębiowski et al., 2008; Gołębiowski et al., 2011; Gołębiowski et al., 2012a; Gołębiowski et al., 2013a; Gołębiowski et al., 2014a; Gołębiowski et al., 2014b; Gołębiowski et al., 2015; Bogus et al., 2010; Bogus et al., 2017; Urbanek et al., 2012; Gutierrez et al., 2015; Paszkiewicz et al., 2016a; Wrońska et al., 2018; Kaczmarek et al., 2020a; Cerkowniak et al., 2020; Wojciechowska & Gołębiowski, 2020; Gołębiowski, Bojke & Tkaczuk, 2021; Kaczmarek & Bogus, 2021a; Kaczmarek & Boguś, 2021b; Kazek et al., 2021). Therefore, an understanding of the FFA profile could assist in the identification and control of insect pests. In addition, during their lifespan, the insect demonstrates changes in lipid composition related to its stage of development; these changes are influenced by body composition, lifestyle, diet, sex and the environment (Lease & Wolf, 2011; Gołębiowski, 2012; Gołębiowski et al., 2014b; Gołębiowski et al., 2016; Toprak et al., 2014; Kazek et al., 2019; Ewald et al., 2020; Seite et al., 2021; Kaczmarek et al., 2021).

### Why the study needed

The past decades have seen a growth in interest in entomological physiology and morphology. Insects are important vectors of disease, causing infection via wounds and necrotic tissue; they can also act as parasitoids and predators, and can contribute to losses in agriculture. However, they also have potential medical value; for example, maggots can be used to treat gangrene and wounds as *maggot therapy*, a procedure approved by the Food and Drug Administration (FDA). Insects are also commonly used in forensic science; their presence can indicate the minimum post-mortem interval and provide valuable information about the movement of the body, cause of death, drug use, or poisoning. They have also been proposed as model organisms in research, as a replacement for mammal systems.

Lipid metabolism plays a crucial role in insects. As in other organisms, lipids perform various functions in insects, such as forming constituents of cellular structures, acting as signalling messengers, and serving as the most significant form of stored energy. These lipid reserves are fundamental in certain situations of high metabolic demand, such as flight and egg production. Also, the cuticle lipids, a complex mixture of hydrocarbons, wax esters and acylglycerols, play an essential role in insect fitness, both as a waterproof barrier and a means to regulate the penetration of pesticides and microorganisms; moreover, they also participate in chemical communication events.

Our present work describes the role of FFAs in cold adaptation, as a supply material during development, and as a resistance/susceptibility factor. It also reviews recent scientific reports on FFA transport, which is an important issue concerning all insects.

### Who it is intended for

Our review is intended for researchers hoping to describe insect physiology and biochemistry; however, it includes aspects of insect biology that are relevant for those studying Entomology in general: it describes the role of FFAs in cold adaptation, as a supply material during holometabolous development, and as a resistance or susceptibility factor, and reviews recent scientific reports on FFA transport, which is applicable to all insects. In addition, insects can be used as models, which can be a potential alternative for researchers using vertebrate research models. Finally, our findings may be of interest for researchers studying the interactions between hosts and pathogens with the aim of creating innovative and effective biocontrol agents.

### Survey Methodology

A range of approaches were to identify articles. Firstly, a search was performed on popular platforms such as Google Scholar and PubMed. The popular keywords were *insect*, *free fatty acid* and *holometabolous/hemimetabolous/hemimetabolous, hematophagous, development, adaptation to cold, oleic acid, linoleic acid, thermal compensation - rapid cold-hardening (RCH), diapause, metabolism of lipids, transport of lipids, lipophorin, HDLp, LDLp, VHDLp, plasma membrane fatty acid binding protein (FABPpm), fatty acid translocase (FAT/CD36), fatty acid transport proteins (FATPs)*, *Acetyl-CoA carboxylase (ACC)*, *Fatty acid synthase (FAS), Very long-chain fatty acid elongase (ELOVL), Long-chain acyl-CoA synthetase (ACSL) Fatty acid desaturase (FAD), free fatty acid as a resistance factor* The search was refined by re-arranging the keywords as necessary. The search only included articles in English. After a large number of articles was identified, they were finalized for the review process by reading the abstract; following this, articles the most relevant to the topic were chosen for further analysis. Some papers regarded as having a good research contribution was further scrutinised to identify further references.

### Classification of FFAs

FFAs can be classified into several groups with respect to their structure, physiological role and biological effects. The classification and examples of FFAs are presented in Table 1

**Table 1:** Classification of FFAs with examples in insects.

	**Definition**	**Examples of FFAs** **(Numerical symbol; systematic name; common name; chemical structure)**		**Examples in Incects**		**Literature data**
**Length of FAs**				
**Short-chain fatty acids (SCFAs)**	up to 5 carbon atoms	C2:0; Ethanoic acid; Acetic acid; CH_3_COOH C3:0; Propionic acid; Propanoic acid; CH_3_CH_2_COOH C4:0; Butyric acid; Butanoic acid; CH_3_(CH_2_)_2_COOH		*Apis mellifera* gut microbiota - *Gilliamella apicola*		Zheng et al. (2017b)
**Medium-chain fatty acids (MCFAs)**	6 to 12 carbon atoms	C6:0; Hexanoic acid; Caproic acid; CH_3_(CH_2_)_4_COOH C8:0; Octanoic acid; Caprylic acid; CH_3_(CH_2_)_6_COOH C10:0; Decanoic acid; Capric acid; CH_3_(CH_2_)_8_COOH C12:0; Dodecanoic acid; Lauric acid; CH_3_(CH_2_)_10_COOH		*Hermetia illucens*, *Oecophylla smaragdina Zophobas morio*, *Tenebrio molitor*, *Gryllus bimaculatus*		Ewald et al. (2020); Jayanegara et al. (2020)
**Long-chain fatty acids (LCFAs)**	13 to 21carbon atoms	C14:0; Tetradecanoic acid; Myristic acid; CH_3_(CH_2_)_12_COOH C16:0; Hexadecanoic acid; Palmitic acid; CH_3_(CH_2_)_14_COOH C18:0; Octadecanoic acid; Stearic acid; CH_3_(CH_2_)_16_COOH		*Tenebrio molitor*		Paul et al. (2017); Zheng et al. (2017a)
				*Blatella germanica*		Pei et al. (2021)
				*Chilecomadia valdiviana*		Herrera, Barros-Parada & Bergmann (2019)
**Very long chain fatty acids (VLCFAs)**	more than 22 carbon atoms	C24:0; Tetracosanoic acid; Lignoceric acid; CH_3_(CH_2_)_22_COOH C26:0; Hexacosanoic acid; Cerotic acid; CH_3_(CH_2_)_24_COOH, C28:0; Octacosanoic acid; Montanic acid; CH_3_(CH_2_)_26_COOH C30:0; Triacontanoic acid; Melissic acid, CH_3_(CH_2_)_28_COOH C32:0; Dotriacontanoic acid, Lacceroic acid, CH_3_(CH_2_)_30_COOH C34:0 : Tetratriacontanoic acid; Ghedoic acid; CH_3_(CH_2_)_32_COOH C35:0: Pentatriacontanoic acid; Ceroplastic acid; CH_3_(CH_2_)_33_COOH		*Tenebrio molior*		Zheng et al. (2017a)
				*Coptotermes formosanus*		Chen, Henderson & Laine (1999)
				*Blatta orientalis*		Gutierrez et al. (2015)
				*Drosophila melanogaster*		Wicker-Thomas et al. (2015)
				*Apis mellifera*		Bharathwaaj et al. (2018)
**Saturation**				
**Saturated**	no C=C double bonds	C16:0; Hexadecanoic acid, Palmitic acid; CH_3_(CH_2_)_14_COOH C18:0: Octadecanoic acid; Stearic acid; CH_3_(CH_2_)_16_COOH		*Galleria mellonella*		Wrońska et al. (2018)
				*Sarcophaga argyrostoma*		Kaczmarek et al. (2020b),
				*Blatta orientalis, Blattella germanica*		Paszkiewicz et al. (2016b); Paszkiewicz et al. (2016a); Kaczmarek et al. (2020b)
				*Calliphora vicina*		Gołębiowski et al. (2012a); Gołębiowski et al. (2012b); Kazek et al. (2021)
				*Lucilia sericata*		Gołębiowski et al. (2012a)
				*Aedes aegypti*		Kaczmarek et al. (2021)
				*Dermestes ater*		Cerkowniak et al. (2020)
**Unsaturated**	one or more C=C double bonds			
**cis**	the two hydrogen atoms adjacent to the double bond stick out on the same side of the chain	C16:1, cis- Δ9, n −7 (9Z)-hexadec-9-enoic acid; Palmitoleic acid CH_3_(CH_2_)_5_CH=CH(CH_2_)7COOH		*Anopheles messeae, Ochlerotatus caspius, O. flavescens, O. euedes, O. cataphylla, Aedes cinereus*		Gladyshev et al. (2011)
**trans**	the adjacent two hydrogen atoms lie on opposite sides of the chain	C18:2; trans,trans- Δ9, Δ12; (9E,12E)-Octadeca-9,12-dienoic acid; Linolelaidic acid; CH_3_(CH_2_)_4_CH=CHCH_2_CH=CH(CH_2_)_7_COOH		*Tenebrio molitor*		Benzertiha et al. (2019)
				*Trachyderma philistina*		(Nancy Taha Mohamed PhD, 2020)
**monounsaturated fatty acids (MUFAs)**	One C=C double bonds	C16:1; cis- Δ9, n −7 (9Z)-hexadec-9-enoic acid; Palmitoleic acid CH_3_(CH_2_)_5_CH=CH(CH_2_)7COOH C18:1, cis- Δ9, n −9 (9Z)-Octadec-9-enoic acid: Oleic acid CH_3_(CH_2_)_7_CH=CH(CH_2_)_7_COOH				Michaud & Denlinger (2006); Raksakantong et al. (2010); Blaul et al. (2014); McAfee et al. (2018); Tang et al. (2019)	
**polyunsaturated fatty acids (PUFAs)**	More than one C=C double bonds	C18:2, cis,cis- Δ9, Δ12, n −6; (9Z,12Z)-Octadeca-9,12-dienoic acid; Linoleic acid CH_3_(CH_2_)_4_CH=CHCH_2_CH=CH(CH_2_)_7_COOH C18:3, cis,cis,cis- Δ9, Δ12, Δ15, n-3;(9Z,12Z,15Z)-Octadeca-9,12,15-trienoic acid α- Linolenic acid; CH_3_CH_2_CH=CHCH_2_CH=CHCH_2_CH=CH(CH_2_)_7_COOH C20:4; cis,cis,cis,cis- Δ5 Δ8, Δ11, Δ14, n-6 (5Z,8Z,11Z,14Z)-Icosa-5,8,11,14-tetraenoic acid; Arachidonic acid; CH_3_(CH_2_)_4_CH=CHCH_2_CH=CHCH_2_CH=CHCH_2_CH=CH(CH_2_)_3_COOH C20:5, cis,cis,cis,cis,cis- Δ5, Δ8, Δ11, Δ14, Δ17, n −3 (5Z,8Z,11Z,14Z,17Z)-Icosa-5,8,11,14,17-pentaenoic acid: Eicosapentaenoic acid CH_3_CH_2_CH=CHCH_2_CH=CHCH_2_CH=CHCH_2_CH=CHCH_2_CH=CH(CH_2_)_3_COOH				Stanley-Samuelson & Dadd (1983); Blomquist, Borgeson & Vundla (1991); Guil-Guerrero et al. (2018); Hasan, Ahmed & Kim (2019); Vatanparast, Lee & Kim (2020); Scharnweber et al. (2020); Stanley & Kim, (2020); Broschwitz et al. (2021); Duarte et al. (2021); Kim & Stanley (2021); Nishidono et al. (2021)	
**Length of FFAs**				
**Short-chain fatty acids (SCFA)**	up to 5 carbon atoms	C2:0 Ethanoic acid Acetic acid CH_3_COOH C3:0 Propionic acid Propanoic acid CH_3_CH_2_COOH C4:0 Butyric acid Butanoic acid CH_3_(CH_2_)_2_COOH		honeybee (*Apis mellifera*) gut microbiota - Gilliamella apicola (Zheng et al. 2017a)
**medium-chain fatty acids (MCFA)**	6 to 12 carbon atoms	C6:0 Hexanoic acid Caproic acid CH_3_(CH_2_)_4_COOH C8:0 Octanoic acid Caprylic acid CH_3_(CH_2_)_6_COOH C10:0 Decanoic acid Capric acid CH_3_(CH_2_)_8_COOH C12:0 Dodecanoic acid Lauric acid CH_3_(CH_2_)_10_COOH		*Hermetia illucens* maggots, *Oecophylla smaragdina* krotos, *Zophobas morio* superworms, *Tenebrio molitor* mealworms, and *Gryllus bimaculatus* crickets (Ewald et al. (2020); Jayanegara et al. (2020))
**long-chain fatty acids (LCFA)**	13 to 21carbon atoms,	C14:0 Tetradecanoic acid Myristic acid CH_3_(CH_2_)_12_COOH C16:0 Hexadecanoic acid Palmitic acid CH_3_(CH_2_)_14_COOH C18:0 Octadecanoic acid Stearic acid CH_3_(CH_2_)_16_COOH		Tenebrio molitor L (Paul et al. 2017; Zheng et al. 2017b), B. germanica (Pei et al. 2021)), *Chilecomadia valdiviana* (Herrera, Barros-Parada & Bergmann, 2019)
**very long chain fatty acids (VLCFA)**	more than 22 carbon atoms	C24:0 tetracosanoic acid lignoceric acid CH_3_(CH_2_)_22_COOH C26:0 Hexacosanoic acid cerotic acid CH_3_(CH_2_)_24_COOH, C28:0 Octacosanoic acid montanic acid CH_3_(CH_2_)_26_COOH C30:0 Triacontanoic acid melissic acid CH_3_(CH_2_)_28_COOH C32:0 Dotriacontanoic acid lacceroic acid CH_3_(CH_2_)_30_COOH C34:0 Tetratriacontanoic acid ghedoic acid CH_3_(CH_2_)_32_COOH C35:0 Pentatriacontanoic acid ceroplastic acid CH_3_(CH_2_)_33_COOH		*Tenebrio molior* (Zheng et al. 2017b), *Coptotermes formosanus* (Chen, Henderson & Laine, 1999), *B. orientalis* (Gutierrez et al., 2015), *Drosophila melanogaster* (Wicker Thomas et al. 2015), *Apis mellifera* (Bharathwaaj et al. 2018)
**Saturation**				
**Saturated**	no C=C double bonds	C16:0 Hexadecanoic acid Palmitic acid CH_3_(CH_2_)_14_COOH C18:0 Octadecanoic acid Stearic acid CH_3_(CH_2_)_16_COOH		(Gołębiowski et al., 2008; Gołębiowski et al., 2012a; Gołębiowski et al., 2012b; Wrońska et al. 2018; Kaczmarek et al., 2020a; [Bibr ref-178][Bibr ref-177])
**Unsaturated**	one or more C=C double bonds			
**cis**	the two hydrogen atoms adjacent to the double bond stick out on the same side of the chain			
**trans**	the adjacent two hydrogen atoms lie on opposite sides of the chain			
**monounsaturated fatty acids (MUFAs)**	One C=C double bonds	C16:1, cis-Δ9, n–7 (9Z)-hexadec-9-enoic acid Palmitoleic acid CH_3_(CH_2_)_5_CH=CH(CH_2_)7COOH C18:1, cis-Δ9, n–7 (9Z)-Octadec-9-enoic acid Oleic acid CH_3_(CH_2_)_7_CH=CH(CH_2_)_7_COOH		(Michaud & Denlinger, 2006; Raksakantong et al. 2010; Blaul et al. 2014; McAfee et al. 2018; Tang et al. 2019)
**Polyunsaturated fatty acids (PUFAs)**	More than one C=C double bonds	C18:2, cis,cis-Δ9,Δ12, n–6 (9Z,12Z)-Octadeca-9,12-dienoic acid Linoleic acid CH_3_(CH_2_)_4_CH=CHCH_2_CH=CH(CH_2_)_7_COOH C18:3, cis,cis,cis-Δ9,Δ12,Δ15, n–3 (9Z,12Z,15Z)-Octadeca-9,12,15-trienoic acid *α*- Linolenic acid CH_3_CH_2_CH=CHCH_2_CH=CHCH_2_CH=CH(CH_2_)_7_COOH C20:4, cis,cis,cis,cis-Δ5Δ8,Δ11,Δ14, n–6 (5Z,8Z,11Z,14Z)-Icosa-5,8,11,14-tetraenoic acid Arachidonic acid CH_3_(CH_2_)_4_CH=CHCH_2_CH=CHCH_2_CH=CHCH_2_CH=CH(CH_2_)_3_COOH C20:5, cis,cis,cis,cis,cis-Δ5,Δ8,Δ11,Δ14,Δ17, n–3 (5Z,8Z,11Z,14Z,17Z)-Icosa-5,8,11,14,17-pentaenoic acid Eicosapentaenoic acid CH_3_CH_2_CH=CHCH_2_CH=CHCH_2_CH=CHCH_2_CH=CHCH_2_CH=CH(CH_2_)_3_COOH		Stanley-Samuelson & Dadd (1983); Blomquist, Borgeson & Vundla, 1991; Guil-Guerrero et al. (2018); Hasan et al. (2019); Vatanparast et al. (2020); Scharnweber et al. (2020); Stanley & Kim (2020); Broschwitz et al. (2021); Duarte et al. (2021); Kim & Stanley (2021); Nishidono et al. (2021)

Fatty acids have a backbone made of carbon atoms and they vary in the number of carbon atoms, and in the number of double bonds between them. Short-chain fatty acids (SCFAs) are fatty acids with up to 5 carbon atoms, medium-chain fatty acids (MCFAs) have 6 to 12, long-chain fatty acids (LCFAs) 13 to 21, and very long chain fatty acids (VLCFAs) are fatty acids with more than 22 carbon atoms. Fatty acids are classified according to the presence and number of double bonds in their carbon chain. Saturated fatty acids (SFAs) contain no double bonds, monounsaturated fatty acids (MUFAs) contain one, and polyunsaturated fatty acids (PUFAs) contain more than one double bond. Unsaturated fatty acids can also be classified as *cis* (bent form) or *trans* (straight form), depending on whether hydrogen is bound on the same, or on the opposite side of the molecule. Most naturally occurring unsaturated fatty acids are found in cis form. Trans fatty acids (TFAs) can be divided in two groups: artificial TFAs (industrial) and natural TFAs (ruminant) (Tvrzicka et al., 2011; Yoon et al., 2018).

### FFAs during the development of insects

#### Holometabolous development

Lipid accumulation and mobilisation also plays particularly important roles in the radical reconstruction of body plan and biochemistry in holometabolous insects. Holometabolous metamorphosis is characterised by four developmental stages: egg, larva, pupa and adult. While most females lay eggs and are thus oviparous, Sarcophagidae might be larviparous, meaning that the egg develops internally, and the females give birth to first-instar larvae. A few insect groups, such as louse flies (Hippoboscidae) and tsetse flies (Glossinidae), are pupiparous: the developing larvae are retained within the maternal body until they are ready to pupate. Larviparous and pupiparous species produce lower numbers of offspring per female compared with oviparous and ovoviviparous species (Gerhardt & Hribar, 2019).

Holometabolous insects have the advantage that they develop though specialised stages: the larva for feeding and growth, and adult form for reproduction (Horne, Haritos & Oakeshott, 2009). Moreover, larvae and adults do not compete for the same food, because they have been adapted to different ecological niches (Terra & Ferreira, 2020).

The lipid content of holometabolous insects increases steadily during larval development to match the metabolic requirements of the larva, and allows the accumulation of energy reserves for metamorphosis (Lease & Wolf, 2011). Nutritional deficiencies during larval feeding and development result in smaller adult size, lower resistance to environmental stress and reduced reproductive capacity (Nestel et al., 2016; Pascacio-Villafan et al., 2016); in addition, in the Lepidoptera, lipid deficiencies during the larval stage might result in incorrect male behaviour, decreased reproductive investment (Vande Velde, Schtickzelle & Van Dyck, 2013) and adult fitness (Boggs & Freeman, 2005), as well as compromised immune system function and pathogen resistance (Lee et al., 2006a).

The fatty acid content of black soldier fly larvae *Hermetia illucens* (Diptera: Stratiomyidae), which are able to feed on a variety of organic materials and are considered alternative sources of both protein and fat in feeds used in aquaculture (Diener, Zurbrugg & Tockner, 2009; Wang & Shelomi, 2017), is strongly correlated with diet and larval weight; larvae with a higher weight contained a higher percentage of saturated fatty acids and a lower percentage of unsaturated fatty acids, such as eicosapentaenoic (EPA) and docosahexaenoic acid (DHA) (Li et al., 2016; Ewald et al., 2020). The different fatty acid profiles of black soldier flies and larvae and prepupae may be related to the modulation of lipid metabolism-related gene expression during larval development (Giannetto et al., 2020).

During metamorphosis, most larval tissues degenerate and adult structures are synthesized *de novo* from imaginal discs (Truman & Riddiford, 2019). This process is dependent upon the levels of energy reserves, such as lipids, and precursors accumulated during larval growth. The presence of lower amounts of internal FFAs during the pupal stage might be connected with the disintegration of the larval fat body in the pupal stage (Kurata, Komano & Natori, 1989). In addition, chemical analyses of cuticular compounds, including FFAs, in insects and other arthropods are often used to estimate the post-mortem index in criminal investigations, and are considered substitutes for traditional morphological examinations and/or DNA-based methods (Gołębiowski et al., 2014a; Kranz et al., 2017; Kaczmarek et al., 2020b).

The cuticular lipid content in the imago varies according to age. Research on *Sarcophaga bullata* (Diptera: Sarcophagidae*)*, has shown that FFAs constitute 26% all cuticular lipids in newly emerged flies and 45% in seven-days-old and suggest that lipid synthesis or transport to the cuticle surface is incomplete in newly-emerged adults. There are also morphological differences of the cuticles of newly-emerged adults, which are pliable and paler coloured then older ones; transport of FFAs occurs primarily through the unhardened cuticle, and this process is essentially completed by the time the adult cuticle is fully hardened (Jackson, Armold & Regnier, 1974). Also, in *Drosophila* females, the lipid content increased with age , and this correlates with increased resistance to starvation (Service, 1987).

Adult insects that do not feed rely on these reserves to support life and reproduction; however, some holometabolous insects, such as blood-feeding mosquitos, require meals to increase protein or lipid levels to enable, or even enhance, reproductive success (Arrese & Soulages, 2010). Egg development involves a substantial mobilization of reserves from the fat body to the ovaries. Lipids are potent sources of energy for oocyte maturation in females and also account for the formation of membranes; for example, in the mosquito *Culex quinquefasciatus* (Diptera: Culicidae), ∼90% of the energy used by the developing embryo originates from lipids (Van Handel, 1993) and in tobacco hornworm *Manduca sexta* (Lepidoptera: Sphingidae), a novel host model for the study of fungal virulence and drug efficacy, lipids represent around 40% of the dry weight of a mature oocyte (Soulages & Wells, 1994; Lyons et al., 2020). Although oocytes are able to synthesize fatty acids *de novo*, this contribution does not account for more than 1% of egg lipid content (Arrese & Soulages, 2010) and the vast majority of the lipid accumulated in oocytes originates in the fat body and is transported to the ovaries by lipophorin (Ziegler & Van Antwerpen, 2006; Santos et al., 2011; Lu et al., 2018).

In *Aedes aegypti* (Diptera: Culicidae*)*, feeding females with sugar before a blood meal resulted in lipid accumulation in the fat body, suggesting that *de novo* lipogenesis was active in these insects; in addition, amino acids derived from the blood meal were also used for *de novo* lipogenesis, which improved oogenesis efficiency (Ziegler & Ibrahim, 2001; Zhou, Pennington & Wells, 2004). Research demonstrated that lipid metabolism in the fat body of female *Ae. aegypti* during vitellogenesis is an extremely dynamic process (Pinch et al., 2021). Studies have shown that lipid levels in the fat body of female mosquitoes decrease significantly over the first 36 h post blood meal (PBM) (Hou et al., 2015; Wang et al., 2017). This decrease corresponded with a decrease in lipid droplet size (Wang et al., 2017). In turn, another study found maximal lipid accumulation in the developing oocytes at 30 h PBM (Briegel, Gut & Lea, 2003). These accumulated lipids constitute up to 30–40% of oocyte dry weight (Troy, Anderson & Spielman, 1975; Briegel, 1990; Van Handel, 1993).

The adults of several parasitoid species, such as parasitoid wasps, which are also considered as indicators in forensic science (Rivers, 2016), demonstrate little possibility to accumulate energy supplies and were believed to have no enzymes which synthesize fatty acids *de novo* (Visser & Ellers, 2008). Due to their parasitic lifestyle, parasitic wasps were assumed to receive sufficient amounts of lipids from their hosts during larval development making the ability to synthesize fatty acids redundant, finally leading to the loss of the evolutionary trait (Visser et al., 2010). However, recent studies have shown that wasps from the *Nasonia* goup are able to synthetise palmitic and stearic acid from α-D-glucose (Prager, Bruckmann & Ruther, 2019; Ruther, Prager & Pokorny, 2021) and linoleic acid from oleic acid (Broschwitz et al., 2021). This confirms that these insects possess the enzymatic machinery to synthesize not only the primary products of fatty acid biosynthesis, but also to introduce double bonds at positions 9 and 12 to produce oleic acid and linoleic acid; more importantly, it is not the case that these insects lack lipogenesis due to environmental compensation, as suggested by several previous studies (Visser et al., 2010; Visser et al., 2012).

### Hemimetabolous insects

The life cycle of hemimetabolous insects includes three developmental stages: egg, nymph (several instars) and adult. The early-stage of hemimetabolous insects is characterised by proportionally lower lipid contents than adults, which is contrary situation than in holometabolous insects. The differences in lipid content in immature and adults occur in holometabolous and hemimetabolous insects might be connected with different morphological changes occurs in the growth and reproduction phase in both insect insects (Lease & Wolf, 2011). In holometabola the intense periods of growth and the development of reproductive, in other word periods of energy accumulation and use is observed, and thus affect energy storage. In hemimetabolous insects, this kind of shifts are observed also, however there are not direct changes from an immature insect to an adult insect (Chapman, 1998). On the other hand, metamorphosis in the holometabola includes a stage characterised by the breakdown of larval tissue and formation of adult tissue; this nonfeeding stage is fuelled completely by energy reserves, which accumulate steadily during larval development (Downer & Matthews, 1976; Lease & Wolf, 2011). This abrupt transition between energy accumulation (as a larva) and energy use (as a pupa, preparing to be an adult) might underlie the higher relative lipid content of holometabolous larvae compared with hemimetabolous larvae (Lease & Wolf, 2011).

A higher FFA content was also observed for adults than nymphs for cockroaches: *Blatta orientalis, Blaptica dubia* (both Blattodea: Blaberidae), *Blatella germanica*, *Blaberus discoidalis* and *Blatta lateralis* (all Blattodea: Blaberidae) (Gutierrez et al., 2015; Kulma et al., 2016) and the highest concentration was observed for C16:0, C18:1, C18:2 and C18:0 in both development stage (Gutierrez et al., 2015; Kulma et al., 2016; Paszkiewicz et al., 2016a; Paszkiewicz et al., 2016b; Kaczmarek et al., 2020a). Moreover, the different distribution of cuticular FFAs in wings and thoraces of adults *B. germanica* and *B. orientalis* was observed. Although these species have similar whole body FFA content, the higher concentration of FFAs in wings and thoraces was observed in *B. germanica* than in *B. orientalis* (Kaczmarek et al., 2020a).

The metabolism of dietary lipids is important for kissing bugs, as it allows the storage of lipid resources in peripheral tissues. After large blood meal the fifth-instar nymphs of *Panstrongylus megistus* (Hemiptera: Reduviidae) control the digestion of lipid process. The few days after the blood meal the regulation of mobilization of lipids from the lumen and consequently, their transfer to the hemolymph and storage in fat body is still well-regulated (Canavoso, Frede & Rubiolo, 2004). The ingested triglicerides (TAGs) are hydrolysed to FA in the *P. megistus* midgut lumen (Canavoso, Frede & Rubiolo, 2004). The lipid loading of lipophorin at the midgut is connected with the nutritional status of the insect, being mediated by specific binding sites located at the cell membrane of enterocytes (Fruttero, Rubiolo & Canavoso, 2009).

The lipid transfer to developing oocytes of *P. megistus* is accomplished by endocytosis of lipophorin, and by the classic extracellular lipophorin-shuttle mechanism. In addition, lipids in the oocyte lipid droplets can originate from endocytosed vitellogenin (Fruttero et al., 2011). Considering that in *P. megistus*, lipophorin only constitutes about 3% of the total protein content of ovarian tissue, while lipids comprise almost 40% of the dry weight of a mature egg, it is possible that most of the lipids will be transferred from lipophorin to the oocytes by a selective mechanism operating at the cell surface without lipoprotein internalization. In addition, since significant vitellogenin endocytosis occurs during vitellogenesis, its role in lipid deposition in oocytes should not be disregarded (Fruttero et al., 2011).

The hematophagous hemimetabolous insect, *Rhodnius prolixus* (Hemiptera: Reduviidae) feeds exclusively on large and infrequent blood meals. After feeding, the adult female slowly digests the blood for approximately 14 days, providing diet-derived lipids that are transported through the hemolymph to the fat body and ovary (Grillo, Majerowicz & Gondim, 2007). In the fat body, the amount of stored lipid increases during the first days after a blood meal, decreasing only after approximately 15 days, when digestion is almost complete (Pontes et al., 2008). Blood meal also triggered *de novo* lipid synthesis in fat body of *R. prolixus* (Saraiva et al., 2021). The storage of TAGs in fat body cells is promoted by insulin receptors (IR). The knockdown of RhoprIR (*R. prolixus* IR) resulted in a lower potential to utilize hemolymph-derived fatty acids, which could reduce the levels of TAG and lipid droplet sizes in the fat body and, in turn, impair ovary development and reproductive capacity (Silva-Oliveira et al., 2021). Oocytes accumulate lipids and other yolk components during the meal-induced oogenesis cycle (Gondim, Oliveira & Masuda, 1989; Santos et al., 2008; Santos et al., 2011) and lipids that were incorporated into the ovaries were primarily used to synthesize TAG, which in insect oocytes are stored in lipid droplets (Ziegler & Van Antwerpen, 2006). After oviposition in eggs of *R. prolixus*, the TAG content (70.4%) was slightly higher than in mature oocytes (60%), while the diacylglycerol (DAG) content (9.6%) was lower than in 2.0 mm oocytes (13.9%). These differences illustrated a reorganisation of lipid composition in the later stages of oocyte maturation, after chorion deposition, where about 40% of TAG reserves is used during embryogenesis (Santos et al., 2011). This result, that less than half of the TAG content in the egg was consumed before hatching was similar to that of eggs of other arthropods, where a large portion of the egg lipids are still present in the hatchlings, for example in *C. quinquefasciatus*, where 46% of lipids are consumed during embryogenesis (Van Handel, 1993). On the seventh day after oviposition, about half of the TAG in the egg was associated with the embryo, which is the same amount of lipids found in newly hatched nymphs. In *R. prolixus* part of the substances storage in the yolk, *i.e.,* proteins, glycogen and lipids, are detected in the digestive tract of the nymphs and are used as a nutrients during the initial days after hatching (Santos et al., 2008; Santos et al., 2011). The research on unfed nymph of *R. prolixus* has shown that fifteen days after emergence, about 30% of the TAG originated from the egg is still present, and describes that after hatching, lipids found in the nymph are slowly used by the insect in an efficient and controlled mechanism (Santos et al., 2011).

In *Dipetalogaster maxima* (Hemiptera: Reduviidae) oenocytes, the highest amounts of FFAs were detected at both stages of atresia. Taking into account that follicular atresia involves remarkable changes in both ovarian morphology and oocyte resorption, it is likely that FFAs detected at atretic stages arose from the breakdown of TAG reserves. The extent to which such FFAs provide energy to support the instauration and progression of the degenerative process or recycle to circulation remains to be established (Leyria et al., 2014). Research pointed the role of juvenile hormone in mediation in lipid transfer and storage in the oocytes of *D. maxima* (Ramos et al., 2021) and also speculated about possibility of bidirectional lipophorin-mediated transfer in insect from the oocyte to the hemolymph during atresia. Moreover, the role of lipophorin oocyte receptors seems be crucial in this process, especially that some results suggest that oocyte plasma membrane proteins, unrelated to the low-density lipoprotein receptor (LDLR) superfamily, can function as nonendocytic lipophorin receptors to regulate the lipid transfer process (Leyria et al., 2014).

### FFAs as a fuel for flight

Lipids serve as the main source of energy in long-term flying insects (Briegel, Knusel & Timmermann, 2001; Wang et al., 2009; Lopez et al., 2014). Generally, carbohydrates are used as fuel in the initial stages of flight, with the fuel source being switched to fats during longer flight. The use of carbohydrates is governed by the hormone octopamine, which also increases trehalose concentration in the hemolymph as part of stress responses. During the first few minutes of flight, octopamine also induces the first release of DAG from the fat body, however, the subsequent, more prolonged phase of TAG mobilization occurs through the action of adipokinetic hormone (AKH). As a result, the concentration of DAG in the hemolymph increases and constitutes the principal fuel for flight (Toprak, 2020). Briefly, longer flight stimulates the release of AKH from the corpus cardiacum. AKH acts directly on the fat body and induces lipid mobilization in the adipose (fat body) cells, which ultimately results in the release of DAG from TAG stores (Skowronek, Wójcik & Strachecka, 2021) Adipokinetic Hormone Receptor (AKHR) knockdown leads to TAG accumulation in fat body and flight muscles, and reduced hemolymph lipid levels after starvation in *R. prolixus*, also indicating the requirement of AKHR in TAG mobilization (Zandawala et al., 2015). TAG molecules are transported by lipophorin to flight muscles, where they are digested to FFAs, which are oxidised for energy generation (Van Der Horst et al., 2002; Jenni-Eiermann & Srygley, 2018). The source of energy for flight can differ between insect species, for example Dipteran species, like *D. melanogaster* usually use firstly glycogen as the primary source of energy for flight and then, lipid reserves to maintain long-term flight after several hours (Wigglesworth, 1949; Briegel, Knusel & Timmermann, 2001; Kaufmann & Briegel, 2004), while aphids primarily use lipids (Liquido & Irwin, 1986; Xueke et al., 2017).

Lipids are also involved as an energy source by triatomine bugs during flight. The presence of lipid droplets in flight muscle has been reported in at least three vectors of Chagas disease including *Triatoma infestans* (Hemiptera: Reduviidae), *R. prolixus*, and *D. maxima* (Ward, Candy & Smith, 1982; Cavagnari et al., 2000).

Although *D. maxima* is not a strong flyer, research indicate the presence of fatty acid binding protein (FABP), abundant mitochondria, and lipid stores in the flight muscles, which suggests the presence of beta oxidation of fatty acids as a main energy source for the triatomine thoracic muscle, and could be related to its dispersal capacity (Cavagnari et al., 2000).

Research has shown that the flight in *R. prolixus* induced both changes in the muscle content of lipid and glycogen and the increase of hemolymph lipid. However, no changes in the hemolymph carbohydrate in insects flown for a short time were observed (Ward, Candy & Smith, 1982; Canavoso, Stariolo & Rubiolo, 2003).

Similar changes (decrease of flight muscle lipids, 60% decrease in fat body lipid content and increase lipid level in hemolymph) was observed in *P. megistus* (Hemiptera: Reduviidae) after 45 min of flight (Canavoso, Stariolo & Rubiolo, 2003). The research indicated that the changes in lipid content in hemolymph is connected with low-density lipophorin particle (LDLp), and flight promoted the partial interconversion of lipophorins and therefore, both high-density lipoprotein HDLp and LDLp were observed (Canavoso, Stariolo & Rubiolo, 2003).

### FFAs and adaptation to adverse temperatures

Most insects are ectothermic organisms, for whom physiological sources of heat are of relatively small or quite negligible significance in controlling body temperature and so they gain heat from their environment. Therefore, insects have had to develop mechanisms to allow them to survive in environments with both long-term low temperature variations associated with seasonal changes and short-term daily or local fluctuations. Preventing cellular damage due to low temperatures (cold shock) is a major challenge for insects living in temperate zones. Extended periods of low temperature are one of the conditions for the insect entering a photoperiod-induced diapause (metabolic suppression); to prevent or decrease the effect of cold shock after short-term temperature changes, the insect can use various thermal compensation strategies, such as rapid cold-hardening (RCH), a quick response that can occur at any stage of the life cycle (Michaud & Denlinger, 2006; Zhu et al., 2016).

### Thermal compensation - Rapid cold-hardening (RCH)

The term *thermal compensation* defines processes that allow adaptation to low temperatures while maintaining a high level of metabolism enabling movement and nutrition and allowing the development of the organism.

RCH is a cold-hardening mechanism that overwintering insects possess throughout their lifespan, although the degree of protection imparted by RCH varies with life stage (Chen, Denlinger & Lee, 1987).

The whole-body glycerol content of *Sarcophaga crassipalpis* (Diptera: Sarcophagidae) increases three-fold during RCH, similarly to the level of glycerol in diapausing insect, which suggests that it plays a protective role in this process. Apart from glycerol, various other lipids are also involved in damage prevention. There is known to be a positive correlation between body lipid content and cold tolerance in *Drosophila spp.* (Hoffmann et al., 2001); in addition, Coleman and co-workers, based on a study in which *C. vicina* was fed with a protein-poor diet containing water and sugar, propose that the insect may derive its cold tolerance from its increased lipid content (Coleman, Bale & Hayward, 2015).

FFAs are also an important group involved in cold protection, especially in a process known as homeoviscous adaptation, where the FFA composition is adjusted to maintain the liquid crystalline state at lower temperatures (Sinensky, 1974). Several biochemical mechanisms are employed to help insects survive RCH. For example, membrane fluidity can be enhanced by increasing the proportion of unsaturated fatty acids in the cell membrane, and the ratio of 16-carbon fatty acids relative to 18-carbon fatty acids can be altered, as short-chain fatty acids have lower melting points than long-chain fatty acids; in addition, the position of the moieties within glycerophospholipids can be changed to alter membrane fluidity. Furthermore, the amount of unsaturated and/or short-chain fatty acids in the membrane can be increased, and the polar head groups of membrane phospholipids can be restructured: glycerophosphoethanolamines promote membrane disorder at low temperatures, which also enhances membrane fluidity (Teets & Denlinger, 2013; Teets, Gantz & Kawarasaki, 2020).

In many insects, such as *D. melanogaster*, fast and slow cold hardening causes changes in membrane fatty acid composition, increased fatty acid unsaturation and decreased length of fatty acid chains (Overgaard et al., 2006; Colinet et al., 2016). An increase in the concentration of oleic acid in response to, or in preparation for, low-temperature may maintain proper fluidity of the membrane without sacrificing the delicate balance needed to allow sensitive membrane proteins to continue their optimum function. Increasing proportions of oleic acid have been observed in *Hepialus xiaojinensis* (Lepidoptera: Hepialidae) (Zhu et al., 2016), the ghost moths, which act as hosts for the Chinese caterpillar fungus *Ophiocordyceps sinensis*, which has a long history of use in traditional Chinese medicine (TCM) for its anti-inflammatory and immunomodulatory effects, among others (Zhou et al., 2009; Ng & Wang, 2010; Meng et al., 2015). In *S. crassipalpis*, the pivotal role during RCH and the diapause is played by oleic acid, mostly because is energetically more favorable to manufacture than, for example double bonded fatty acids. It is possible, therefore, that insects that prefer oleic acid in preparation for low temperatures may be preserving finite energy reserves while still gaining the benefit of a wide window of fluidity (Michaud & Denlinger, 2006). In the model organisms for cold research –freeze-tolerant fly *Eurosta solidaginis* (Diptera: Tephritidae) and the freeze-avoiding moth *Epiblema scudderiana* (Lepidoptera: Tortricidae) increased proportions of unsaturated fatty acids during the winter is observed; however, whereas total lipid content did not change in *E. solidaginis*, a decrease in total lipids was observed in *E. scudderiana*, a species sensitive to freezing, over the winter, suggesting the use of reserves to maintain basal metabolism (Joanisse & Storey, 1996).

In *S. crassipalpis*, cold treatment resulted in a 17% increase of total phospholipid fatty acid content; in addition, a decrease of other fatty acids was observed, with the exception of C16:0, which remained the same. These changes are characteristic of RCH; however, in the diapause process, the level of C16:0 changes but C16:1 remains the same (Michaud & Denlinger, 2006). Elsewhere, in *D. melanogaster* has been demonstrated an increase of polyunsaturated linoleic acid and decrease of monounsaturated (18:1) and saturated (14:0) phospholipid fatty acid as a response to RCH. These caused a significant increase in the overall degree of unsaturation and may improve the ability to harden during RCH (Overgaard et al., 2005).

In ghost moth, *H. xiaojinensis*, most unsaturated and long chain FFAs were maintained at higher levels in the hemolymph of cold-acclimated larvae than controls; C16:0 was found to be the most abundant fatty acid in the fat body and the only one whose level changed after cold acclimation, with a reduction of 23%, possibly due to reduced *de novo* synthesis of fatty acids or enhanced transition to other fatty acids (Zhu et al., 2016). Cell membrane fluidity has also been found to increase in response to RCH in the cells of *S. bullata* fat bodies (Lee et al., 2006b). In addition to insects (Khani et al., 2007), faster mobilisation of unsaturated fatty acids compared to saturated acids has been observed in mammals and birds (Raclot, 2003; Price, Krokfors & Guglielmo, 2008) as a general adaptation to cold.

### Diapause

The insect diapause is dynamic state of low metabolic activity, genetically determined, with the neurohormonal system as mediator. Diapause occurs at a species-specific stage of ontogenesis and its expression is regulated by various environmental signals that precede and reliably predict the arrival of unfavourable conditions. The cause–effect relationship between diapause and cold-resistance in arthropods is still debated, and both phenomena may not be directly related, depending on the species and even the population. The main stimulus inducing winter diapause in arthropods is the decrease in day length after summer. Once induced, diapause cannot be immediately terminated even if favourable conditions for development occur (Tougeron, 2019).

The diapause process in *C. vicina* is associated with cold tolerance, and diapausing larvae were more cold tolerant than same-age (nondiapausing) pupae (Saunders & Hayward, 1998). Moreover, *C. vicina* adults exposed to warmer autumn conditions during diapause induction will produce larvae with a reduced cold hardiness capacity, which could negatively impact winter survival. Given that maternal regulation of diapause is common among temperate insects, this could be a widespread phenomenon (Coleman, Bale & Hayward, 2014).

Diapause is characterised by long-term upregulation of heat shock proteins, which prevents protein denaturation and repairs denatured proteins (Hayward et al., 2005), as well as increased glycerol levels. Glycerol acts as an antifreeze agent in insects, lowers the supercooling point of the organism and stabilizes membranes and proteins (Chen, Denlinger & Lee, 1987; Lee et al., 1987; Tsvetkova & Quinn, 1994; Tang & Pikal, 2005). However, it is important to mention that glycerol is not the only antifreeze agent in insects: freeze tolerance is also conferred by trehalose and antifreeze proteins (AFPs). Trehalose is a versatile sugar molecule that can accumulate to high levels in freeze-tolerant and freeze-avoiding insects, where it functions as a cryoprotectant and supercooling agent. Similarly, AFPs lower the freezing temperature and prevent the formation of ice crystals; interestingly, these are present at high levels in both freeze-tolerant insects and some freeze-avoiding insects in winter (Wen et al., 2016).

The process of diapause also influences the amount of lipid reserves in insects, which are used in both catabolic energy production and anabolic processes, including cell membrane maintenance and remodelling (Hahn & Denlinger, 2011). Insects often accumulate lipid stores during diapause-destined or overwintering stages (Hahn & Denlinger, 2011). As most overwintering insects end winter with substantially fewer lipid stores than they began with, it is likely that lipids are a primary source of overwintering fuel (Hahn & Denlinger, 2011).

Diapausing mosquito *Culex pipiens* (Diptera: Culicidae) females accumulate more lipid droplets during diapause preparation and overwintering diapause maintenance than during the nondiapause phase (Zhang et al., 2019). Energy metabolism is crucial for the survival of diapausing insects throughout winter: energy demands are fueled by lipid stores, and *C. pipiens* females begin to utilize TAGs stores, convert stored fat to FFAs, and transport these fatty acids to the mitochondria to meet energetic needs (Zhang et al., 2019). Research has shown that the genes coding for fatty acid synthase-1,-3 and fatty acid binding protein are upregulated, which contributes to the accumulation of TAGs in the fat body (Sim & Denlinger, 2013); after diapause, these lipid reserves are used for egg production (Zhou & Miesfeld, 2009).

The increase of lipid content of diapause compared with non-diapause eggs and 30% more lipid content in larvae entering diapause than their nondiapausing counterparts was observed also for the tiger mosquito, *Aedes albopictus* (Diptera: Culicidae) (Reynolds et al., 2012). The substantial differences in lipid metabolism observed within the embryo occur as a consequence of the diapause program, with some differences taking place both before the actual onset of diapause and during the diapause state. For example, research has shown the elevated expression of the lipid storage droplet protein 2 gene (*lsd2*) during embryonic development, which might likely contribute to the higher amounts of lipid noted in diapausing individuals. The changes in the transcript abundance of genes that regulate lipid storage (*lsd2*), lipolysis (*lip2, lip3,* and *lip4*), *β*-oxidation (*acs, cpt, acd4*, and *acd5*), and unsaturated fatty acid synthesis (*desat*, and *face*), contribute to increased amounts of lipids in diapause embryos. The upregulation of two genes involved in fatty acid synthesis and modification, Δ*(9)-desaturase*, and *fatty acyl-CoA elongase*, was observed in diapausing larvae, which might suggest roles for their gene products in generating unsaturated fatty acids to enhance membrane fluidity at low temperatures and generating precursors to the surface hydrocarbons needed to resist desiccation, respectively (Reynolds et al., 2012).

The gall moth *E. scudderiana* is freeze-avoidant; its lipolysis-related enzyme activity increases in winter, suggesting a shift to lipid metabolism. By contrast, lipolysis enzyme activity decreases over winter in the goldenrod gall fly *E. solidaginis*, a freeze-tolerant species (Joanisse & Storey, 1996). This indicates either a shift to nonlipid fuels or that the rate of lipid metabolism is slowed. However, both species demonstrate an upregulation of 5′adenosine monophosphate-activated protein kinase (AMPK) during winter, implying a shift towards lipolysis (Rider et al., 2011). Flesh flies show the opposite pattern where lipids are depleted early in diapause followed by a shift to other substrates; for example, *S. crassipalpis* larvae destined for pupal diapause contain nearly twice the lipid content as non-diapausing larvae. A rapid decline of lipid content was observed at the beginning of the pupal diapause, but after 40 days, the pupae switched from lipid to nonlipid energy reserves, and the lipid content stabilized and remained nearly constant until adult eclosion (Adedokun & Denlinger, 1985). Research into diapausing *S. crassipalpis* pupae also revealed an increase in the concentration of fatty acid C18:1 in the cell membrane, while those of other fatty acid decreased; however, the level of C16:1 remained the same, and as a consequence, the ratio of unsaturated to saturated fatty acids in diapausing individuals increased (Michaud & Denlinger, 2006), which is a common adaptation to low temperatures in insects (Harwood & Takata, 1965; Valder, Hopkins & Valder, 1969; Bridges & Watts, 1975; Ohtsu et al., 1993; Kostal & Simek, 1998; Tomčala et al., 2006).

The cosmopolitan wasp *Nasonia vitripennis* (Hymenoptera: Pteromalidae), whose broad host range, rate of parasitism and seasonal prevalence afford it great potential as an indicator of time of death in forensic investigations (Voss, Spafford & Dadour, 2009), has been found to contain corresponding levels of glycerol to its host. *N. vitripennis* larvae fed on diapausing *S. crassipalpis* pupae contained four times more glycerol than parasitoids consuming nondiapausing hosts, and the wasp larvae which contained high levels of glycerol also exhibited greater cold tolerance (Rivers, Lee & Denlinger, 2000).

Several hormones have contributed in lipid metabolism during diapause, like AKH, insulin, neuropeptide F (NPF) and short Neuropeptide F (sNPF) or Diapause Hormone-Pheromone Biosynthesis Activating Neuropeptide (DH-PBAN) (Toprak, 2020).

AKHs do not contribute to diapause-associated alterations in metabolism. They are produced in response to AMPH activation in insects, which leads to the release of DAG into hemolymph from TAG stores in the fat body. This takes place via a cyclic cAMP and calcium signalling cascade during diapause maintenance (Sinclair & Marshall, 2018; Toprak, 2020).

Another important hormone involves in diapause is insulin. The relationship between diapause-dependent lipid accumulations and insulin has been shown in *Drosophila* (Tatar & Yin, 2001) and *C. pipiens* (Sim & Denlinger, 2013). Additionally, silencing insulin receptor (InR) in non-diapausing females inhibits ovary development, which simulates the diapause state (Sim & Denlinger, 2008). As expected, the insulin effect occurs mainly during feeding, *i.e.,* before the initiation of the diapause. For example, adult females of *C. pipiens* increase feeding with sugar instead of blood in the prediapause period and accumulate much greater lipid reserves compared to non-diapausing counterparts (Robich & Denlinger, 2005). In this manner, specific insulin-like peptides (ILPs), such as ILP1, contribute to the diapause regulation-related lipid accumulation in *C. pipiens* (Robich & Denlinger, 2005). Notably, juvenile hormones and ecdysone interfere with insulin signalling in terminating diapause (Denlinger, Yocum & Rinehart, 2012).

Lipid metabolism during diapause is also dependent on NPF, a functional homolog of mammalian Orexigenic Neuropeptide Y, and sNPF, which is conserved across protostomes, but not present in vertebrates. These peptides are hormones that inhibit peristalsis and the release of digestive enzymes, and preserve nutrition; they are involved in feeding behaviour, nutritional homeostasis and insulin signalling (Fadda et al., 2019; Toprak, 2020; Skowronek, Wójcik & Strachecka, 2021). In *Drosophila* larvae, it has been found that the accumulation of lipid sources for diapause preparation is dependent *inter alia* on NPF activation: overexpression of NPF leads to prolonged feeding, and NPF-deficient mutant larvae feed less (Fadda et al., 2019).

### Metabolism of FFAs

#### Preference of dietary FFAs by insects

The fatty acid profiles of insects can differ depending on different growth stages, temperatures and nutritional regimes. Insects are able to take up dietary C20:5, which has been found to alter the overall fatty acid profile of tissue phospholipids in lengthy feeding experiments (Stanley-Samuelson & Dadd, 1981; Stanley-Samuelson & Dadd, 1984; Gadelhak et al., 1995). Research has shown that some insect species have dietary preferences regarding FFAs. For example, the adult yellow fever mosquitoes *Ae. aegypti* (Bosch, Geier & Boeckh, 2000), *Anopheles gambiae* (Diptera: Culicidae) (Smallegange et al., 2009), and *T. infestans* (Barrozo & Lazzari, 2004) are attracted by specific FFAs (combined with L-lactic acid); and also, some FFAs might discourage some flies (Mullens, Reifenrath & Butler, 2009) and mosquitoes (Skinner et al., 1970). This preference towards certain FFAs could change during life, for example *D. melanogaster* larvae prefer unsaturated FFAs whereas the adults prefer saturated FFAs (Fougeron et al., 2011).

#### Absorption and storage of lipids

**Figure 1:**
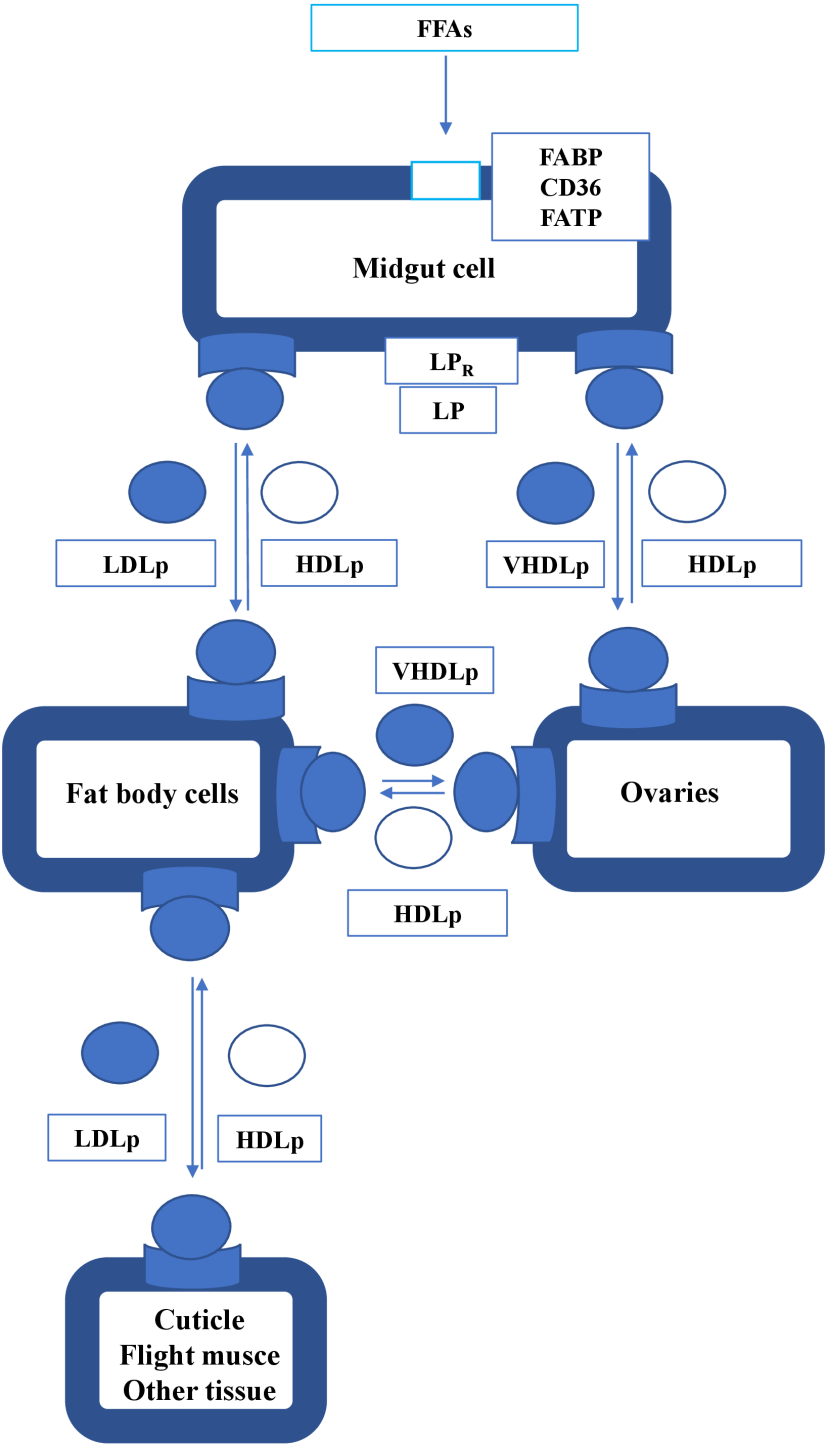
The absorption and transport of FFAs in insect. FFA, free fatty acid; FABP, fatty acid binding protein; FATP, fatty acid transport protein; LP, lipophorin; LP_R, lipophorin receptor; HDLp, high-density lipophorin particle; LDLp, low-density lipophorin particle; VHDLp, very high lipophorin particle.

Lipids are absorbed from food through the middle intestine and transferred from the intestine to the hemolymph in the form of diglycerides (DGs). At this stage, DGs bind to HDL (produced from the apoliprotein in the fat body) and then to LDL. Lipids are transported in the body by lipophorin (Skowronek, Wójcik & Strachecka, 2021). The absorption of FFAs is presented schematically on Fig. 1 The *R. prolixus* gut is compartmentalized into three segments that perform different functions during blood digestion, with fatty acid adsorption taking place in the posterior midgut (Grillo, Majerowicz & Gondim, 2007; Ribeiro et al., 2014). When consumed, DAGs from the hemolymph are bound by LDL and hydrolyzed to fatty acids, which are transported to fat body cells (Grillo, Majerowicz & Gondim, 2007). A different pattern was observed in *P. megistus*, where significant digestion takes place in the anterior midgut (Canavoso, Frede & Rubiolo, 2004).

After absorption, FFAs are immediately bound to binding proteins to avoid toxic effects induced by a rise in intracellular FFA concentration, and to allow their rapid metabolic flow to utilization pathways or storage sites (Glatz, Luiken & Bonen, 2010; Toprak et al., 2020).

Lipids absorbed from the midgut are generally stored in fat body cells. Fat body tissues are major sites for nutrient storage, energy metabolism, innate immunity and detoxification. Recent studies have revealed that the fat body plays a central role in the integration of hormonal and nutritional signals to regulate larval growth, body size, circadian clock, pupal diapause, longevity, feeding behaviour, and courtship behaviour, partially by releasing fat body signals to remotely control the brain. In addition, the fat body has emerged as a fascinating model for studying metabolic disorders and immune diseases (Lehmann, 2018; Li, Yu & Feng, 2019; Toprak, 2020; Toprak et al., 2020; Skowronek, Wójcik & Strachecka, 2021).

The adipocytes, *i.e.,* cells involved in lipid metabolism, are able to store lipid as TAGs, these being strongly hydrophobic neutral lipid droplets with high energy content (Arrese et al., 2001; Arrese & Soulages, 2010; Skowronek, Wójcik & Strachecka, 2021). The ability to mobilize stored TAG reserves is important for the survival of insects under energy-demanding conditions (Lehmann, 2018). The start of lipid mobilisation is indicated by the release of AKH from the corpora cardiaca, which binds to AKH-receptors in adipocytes, leading to the generation of the secondary messenger 3′,5′-cyclic monophosphate (cAMP) and the phospholipase C (PLC) and Ca^2+^ cascades (Toprak, 2020; Toprak, Dogan & Hegedus, 2021). cAMP induces cAMP-dependent protein kinase, leading to activation of the lipolytic transcription factor foxO, which is believed to act on lipase genes (Baumbach et al., 2014); this is accompanied by the hydrolysis of phosphatidylinositol 4,5-bisphosphate (PIP2) to inositol 1,4,5-trisphosphate (IP3) by PLC, which binds to inositol 1,4,5- triphosphate receptor (IP_3_R); this results in activation of the channel and an elevation in cytosolic Ca^2+^ levels (Toprak et al., 2020). Thus, AKH activity results in lipolysis accompanied by an increase in the cytosolic levels of Ca^2+^ in adipocytes (Toprak et al., 2020).

An important aspect of the proper functioning of lipid metabolism is calcium ion homeostasis (Ca^2+^). Many functions in the insect organism, including diapause, metamorphosis, hunger sensing, and receiving signals from neurotransmitters require a certain level of Ca^2+^ to be maintained. In addition, studies have shown that cytosolic Ca^2+^ levels correspond with the levels of triglycerides in lipid droplets, and the process of lipolysis and lipogenesis are dependent on the Ca^2+^ in invertebrates (Skowronek, Wójcik & Strachecka, 2021; Toprak, Dogan & Hegedus, 2021). Most of the data on the involvement of insect endoplasmic reticulum Ca^2+^ channels in lipid metabolism are related to IP_3_R, which induce lipolysis in insect adipocytes (Toprak, Dogan & Hegedus, 2021). The loss of IP_3_R leads to elevated levels of triglycerides with enlarged lipid droplets in the fat body and hyperphagia in *D. melanogaster* adults (Subramanian et al., 2013). In line with this, fat body-specific knockdown of IP_3_R leads to an increase in lipid droplet size and triglyceride accumulation in adult flies (Bi et al., 2014). On the other hand, AKH-induced lipolysis has been reported only in adults of *D. melanogaster* as manipulation of cytosolic Ca^2+^ levels in the larval fat body does not have a significant effect on larval fat stores (Baumbach et al., 2014; Galikova et al., 2015). In contrast, insects, such as *Leptinotarsa decemlineata* (Coleoptera: Chrysomelidae), accumulate greater amounts of lipid at the larval stage, which show impaired lipid metabolism upon silencing Ca^2+^-signaling genes (Doğan et al., 2021; Güney et al., 2021). Therefore, the dynamics of lipid metabolism in relation to Ca^2+^ might be different depending on the species. Dogan et al. investigated the key binding proteins, finding calmodulin, calcineurin, and regucalcin in the *L. decemlineata* fat body. This indicates additional adaptation of this tissue to lipid metabolism (Doğan et al., 2021).

#### Transport of lipids

In insects, lipid transfer to the tissues is mediated by lipophorin, the major circulating lipoprotein (Van der Horst, Roosendaal & Rodenburg, 2009). Lipophorins are synthesised in the fat body and secreted to the circulation. Here, they perform several cycles of lipid loading and unloading, without internalization or further degradation of the apolipoprotein matrix (Fruttero, Leyria & Canavoso, 2017).

Lipophorins were purified from the hemolymph of various insect vectors, including the *T. infestans*, *R. prolixus*, *Glossina morsitans* (Diptera: Glossinidae), *Ae. aegypti, C. quinquefasciatus, Anopheles albimanus* (Diptera: Culicidae), *An. gambiae, Simulium vittatum. Musca domestica* (Diptera: Muscidae) and *Lucilia cuprina* (Diptera: Calliphoridae) (Bianchi, Capurro & Marinotti, 1987; Trowell et al., 1994; Pennington & Wells, 2002; Gondim et al., 2018). The major function and examples of lipophorin are presented in Table 2

**Table 2:** The function of lipophorin and examples of its occurrence in insects.

	**Description**	**Function**	**Examples in insects**	**Literature data**
**HDLp**	high-density lipoprotein/	reach midgut or fat body to acquire fatty acid, delivers pheromones to the cuticle and specialized pheromone glands, hydrocarbons to the epicuticle, fat body, and ovaries and retinoids to yet undetermined locations	*Aedes aegypti*	Capurro, de Bianchi & Marinotti (1994); Cheon et al. (2001)
			*Anopheles gambiae*	Atella et al. (2006)
			*Blattella germanica*	Sevala et al. (1999); Young et al. (1999)
			*Culex quinquefasciatus*	Kumar & Paily (2011)
			*Dipetalogaster maxima*	Leyria et al. (2014)
			*Drosophila melanogaster*	Palm et al. (2012)
			*Glossina morsitans*	Ochanda et al. (1991)
			*Lucilia cuprina*	Trowell et al. (1994)
**LDLp**	low-density lipoprotein	take up lipids released from the midgut/fat body cells and transport them to the fat body or to selectively unload its lipid cargo at target tissues without endocytosis and lysosomal degradation	*Musca domestica*	De Bianchi, Capurro & Marinotti (1987); Schal et al. (2001)
			*Panstrongylus megistus*	Fruttero, Rubiolo & Canavoso (2009); Fruttero et al. (2011)
			*Rhodnius prolixus*	Grillo, Pontes & Gondim (2003); Entringer et al. (2013)
			*Sarcophaga bullata*	Geysen et al. (1988)
			*Simulium vittatum*	Pennington & Wells (2002)
			*Triatoma* *infestans*	Fichera & Brenner (1982)
**VHDLp**	very high-density lipoprotein	transport precursors from the fat body to the ovaries for the deposition of lipid yolk droplets; the constituents of the protein yolk body	*Aedes aegypti*	Sun et al. (2000)
			*Rhodnius prolixus*	Santos et al. (2011)
			*Triatoma infestans*	Rimoldi et al. (1989); Rimoldi et al. (1996)

In *P. megistus*, the process of lipophorin binding in the midgut is dependent on the nutritional status of the insect, with a higher binding activity being observed three days after blood feeding (Fruttero, Rubiolo & Canavoso, 2009). Interestingly, during this period, about 50% of dietary lipids had been digested in the midgut lumen, allowing a significant rate of lipid absorption and transfer to lipophorin (Canavoso, Frede & Rubiolo, 2004). A similar result was observed in *R. prolixus* (Grillo, Pontes & Gondim, 2003), where a very high capacity to bind lipophorin in the midgut was observed on the second day after feeding. The lipophorin binding capacity of the organ decreased and at day 10, when digestion was close to the end (Grillo, Pontes & Gondim, 2003). The high rate of lipid transfer to lipophorin observed in both species suggests that digestion of dietary lipids is an important factor influencing lipophorin binding and consequently, the ability of the tissue to export lipids to the circulation.

The principal circulating lipoprotein in the hemolymph of resting insects is HDLp (1.063–1.21 g/ml), and under physiological conditions characterised by intense energy demands, such as flight or oogenesis, this lipophorin increases its lipid content by binding additional DAG at the surface of the adipocytes. However, mosquito lipophorins have a unique characteristic: they carry TAG rather than DAG as the principle neutral lipid (Ford & Van Heusden, 1994). Either way, the binding of DAG or TAG to the lipophorin results in the production of LDLp, (1.006–1.063 g/ml), which can carry the DAG, or TAG, through the haemolymph to the peripheral tissues (Blacklock & Ryan, 1994; Arrese & Soulages, 2010; Fruttero, Leyria & Canavoso, 2017; Toprak et al., 2020).

Literature data suggests the additional presence of very high-density lipophorin (VHDLp) (Kawooya & Law, 1988; Kawooya, Osir & Law, 1988); this is formed when lipophorin is taken up by oocytes, deposited in yolk bodies, and stripped from its lipids by lipase. This transformation results in the formation of higher density lipophorin particles (∼1.238 g/ml) (Liu & Ryan, 1991; Ziegler & Van Antwerpen, 2006).

The presence of VHDL, a hexameric protein, was detected in various tissues of *T. infestans* throughout the last nymphal and adult stages, and in egg extracts (Rimoldi et al., 1996). Hemolymph VHDL reaches a maximum value before the last moult; it abruptly declines in males and females just after emergence but increases again during adult life. Fat body VHDL decreases slowly and continuously during nymph growth, reaching a minimum value prior to moulting: the values can be as much as two-fold lower in the first week of adult life; its level then follows accumulation and depletion cycles which differ between males and females (Gonzalez, Soulages & Brenner, 1991; Rimoldi et al., 1996).

Most insect lipophorins have two structural apolipoproteins, apolipophorin I (apoLp-I, Mr ∼230–250 kDa) and apolipophorin II (apoLp-II, ∼70–85 kDa) (Fruttero, Leyria & Canavoso, 2017). ApoLp-I and apoLp-II are integral components of the lipophorin particle, and cannot be removed without destruction of lipophorin particle integrity (Van der Horst & Ryan, 2012). It is recognized that apoLp-I and apoLp-II are the product of the same gene, and that the two proteins arise through post-translational cleavage of their common precursor protein (Weers et al., 1993). ApoLp-II/I is a homolog of the mammalian apoB and belongs to the same superfamily of large lipid transfer proteins (LLTP) (Van Der Horst & Rodenburg, 2010).

ApoLp-I and ApoLp-II are responsible for transport of dietary cholesterol in *R. prolixus* (Entringer, Majerowicz & Gondim, 2021). They have been found to mediate lipid transfer to developing eggs and manage the distribution of the imported lipid in developing embryos in *An. gambiae* and *Anopheles aquasalis* (Diptera: Culicidae*)* (Atella et al., 2006; Dias-Lopes et al., 2016). In the tsetse fly *G. morsitans*, RNA interference (RNAi) against GmmapoLp-II/I resulted in decreased hemolymph lipid levels in females and delayed oocyte development (Benoit et al., 2011). In *D. melanogaster*, DmapoLp-II/I was shown to be the major lipid transport protein in the larval hemolymph (Palm et al., 2012). Lipoprotein serves as a carrier for the transport of hydrocarbons from the site of synthesis (oenocyte) to the site of deposition (cuticle) in the American cockroach *Periplaneta americana* (Blattodea: Blattidae) and it is speculated that after synthesis in the oenocytes, the lipids bind to apoLps and are released into the hemolymph; they subsequently shuttle to the epidermis where they bind to lipoprotein receptors, and are finally transported to the cuticle surface via pore canals (Haruhito & Haruo, 1982; Chapman, 1998).

A third apolipoprotein particle is the low molecular weight apolipophorin-III ApoLpIII; Mr 18–20 kDa), homologous to mammalian apoE (Weers & Ryan, 2006), which circulates free in the hemolymph and is taken up by the HDLp when it acquires DAGs from the fat body; ApoLpIII expands the surface of the lipophorin particle during lipid loading (Van der Horst et al., 2002). In insects that use lipid as a fuel for flight, such as *M. sexta*, ApoLpIII is present in adult hemolymph as a lipid-free protein (Van der Horst & Ryan, 2012). The decreased expression of apoLp-III was observed during diapause in the mosquito, *Ae. albopictus* (Reynolds et al., 2012).

ApoLp-III plays also an important role in the innate immune system (Zdybicka-Barabas & Cytrynska, 2013; Staczek et al., 2018; Maravilla et al., 2020). In *G. mellonella* this apolipoprotein stimulates the expression of antimicrobial peptides and proteins, regulates nodulation and opsonization in cellular immunity, and may act as a pattern recognition protein (Wiesner et al., 1997). Moreover, apoLp-IIIGM binds to the surface of gram-negative bacteria, resulting in the deformation of the bacterial membrane (Zdybicka-Barabas et al., 2013; Zdybicka-Barabas et al., 2014).

Apolipophorin-III acts as a positive regulator of *Plasmodium* development in *Anopheles stephensi* (Diptera: Culicidae) and *An. gambiae* (Gupta et al., 2010; Dhawan et al., 2017). Lipophorin and lipophorin receptor genes activation was observed in the *Ae. aegyptii* fat body as a response to *Plasmodium* infection. The fat bodies were found to demonstrate a similar pattern of lipophorin and lipophorin receptor activation on the mRNA level in response to *Plasmodium* infection as observed with Gram-positive bacteria or fungal infections; however, interestingly, Gram-negative bacterial infection did not change the level of transcription of either gene, which might suggest that the Toll/REL1 pathway is involved in the regulation of both *Lp* and *LpRfb* transcripts during immune challenge (Cheon et al., 2006).

The interaction of lipophorin with tissues is mediated by specific receptors and in many insects, Lipophorin receptors have been characterised as members of the low-density lipoprotein receptor gene superfamily (Entringer et al., 2013).

The transported lipids are hydrolysed into FFAs and glycerol at the surface of the recipient cell by a membrane bound lipase, and the FFAs are transported into the cell via fatty acid binding proteins. They can be either hydrolysed to FFAs by membrane-bound lipophorin lipases (Arrese et al., 2001; Sinclair & Marshall, 2018) or taken up by endocytosis of the lipoprotein-DG complex (Rodenburg & Van Der Horst, 2005) In contrast to vertebrates, HDLp is recycled for additional lipid transport in insects; this increases the efficiency of lipid transport and reduces its overall cost (Van Der Horst et al., 2002)

### Lipophorin receptors

In *R. prolixus*, the transfer of lipids from the posterior midgut to lipophorin is mediated by specific binding sites in the midgut cell membrane. The midgut capacity to bind lipophorin varies according to the time after a meal: it is very high during the second day after a meal, when a high rate of lipid transfer to lipophorin is also observed, and the number of lipophorin receptors in midgut cell membranes may be an important mechanism for controlling lipid flux in *R. prolixus* (Grillo, Majerowicz & Gondim, 2007). The ectopic β-chain of ATP synthase (βATPase) was recently described as a possible non-endocytic lipophorin receptor in the anterior midgut of the hematophagous insect *P. megistus* (Fruttero et al., 2014; Fruttero et al., 2017; Fruttero et al., 2019).

In some insects, the lipophorin receptor (LpR), which binds to lipophorin and accepts its lipid cargo, plays an essential role in female fecundity by mediating the incorporation of lipophorin in developing oocytes (Van Der Horst et al., 2002; Ciudad, Belles & Piulachs, 2007; Parra-Peralbo & Culi, 2011; Palm et al., 2012; Lu et al., 2018). The examples of lipophorin receptors in insect are presented in Table 3

**Table 3:** Examples of lipophorin receptors in insects.

**Insect**	**Name**	**Place of detection**	**Literature data**
***Aedes aegypti***	AaLpRov	ovaries	Cheon et al. (2001)
	AaLpRfb	fat body	Seo et al. (2003)
***Blattella germanica***	BgLpR	fat body and ovarian	Ciudad, Belles & Piulachs (2007)
***Culex quinquefasciatus***		fat body	Kumar & Paily (2011)
***Dipetalogaster maxima***	LpR	ovaries	Ramos et al. (2021)
***Drosophila melanogaster***	LpR1, LpR2	muscle	Kim et al. (2021)
***Galleria mellonella***	LpR_Gm_	fat body	Lee et al. (2003)
***Glossina morsitans morsitans***	GmmLpR	midgut, fat body, milk gland, spermatheca and head	Benoit et al. (2011)
***Leucophaea maderae***	LemLpR	ovaries	Tufail et al. (2009)
***Panstrongylus megistus***	β-ATPase	ovaries	Fruttero, Leyria & Canavoso (2017)
*Rhodnius prolixus*	LpR	ovaries	Entringer et al. (2013); Leyria, Orchard & Lange (2020)
		midgut	Grillo, Pontes & Gondim (2003)
		fat body	Pontes, Grillo & Gondim (2002)

The increase in LpR transcript expression during vitellogenesis has been reported in *D. maxima* (Ramos et al., 2021). In *Ae. aegypti* they are two splice variants of LpR gene products specific to the fat body (AaLpRfb) and ovary (AaLpRov) (Cheon et al., 2001; Seo et al., 2003); these two distinct isoforms of lipophorin receptors, which are derived from a single gene, are differentially expressed in oocytes and the fat body (Seo et al., 2003). The expression of the fat body-specific AaLpRfb transcript is restricted to the post-vitellogenic period, during which production of yolk protein precursors is terminated and the fat body is transformed into a storage depot of lipid, carbohydrate and protein (Seo et al., 2003).

In *D. melanogaster*, although the knockdown of LpR did not affect the lipid content of the fat body and hemolymph, it impaired the uptake of neutral lipids in the oocytes and imaginal discs, suggesting an organ-specific role (Parra-Peralbo & Culi, 2011). In *P. megistus* no differences in the expression of the putative lipophorin receptor β-ATPase were observed between fed and fasted insects; however, the nutritional condition influenced the histological features of the fat body (Fruttero et al., 2019).

Different amounts of Lp receptors in the fat body and ovary was also observed in *R. prolixus,* where ^125^I-Lp demonstrated higher specific binding to the oocyte membrane than to the fat body membrane. This outcome might be a result of the increased amount of lipophorin receptors on the oocytes or to differences in the receptor properties between the fat body and oocytes (Entringer et al., 2013).

In *Drosophila*, PUFA are selectively incorporated into the acyl chains of lipophorin phospholipids and effectively transported to the central nervous system by endocytosis, mediated by the lipophorin receptor (Matsuo et al., 2019). In *Drosophila* also, the muscle-specific knockdown of *LpR1* or *LpR2* genes resulted in mitochondrial dysfunction and reduced proteostasis, which contributed to muscle aging (kyeong et al., 2021).

### Intracellular transport of FFA

Import of FFAs by cells can take place by two main mechanisms working in parallel. When the extracellular FFA concentration is high, they often enter the cell by rapid free diffusion, *flip–flopping* across the lipid component of the membrane bilayer along the resulting FFA gradient (Hamilton & Kamp, 1999; Hamilton, 2007). However, in many cell types and organs, transport occurs primarily through a saturable mechanism mediated by FFA-binding protein (Hamilton, 1998; Schaffer, 2002; Doege & Stah, 2006). Three main types of FFA binding proteins are believed to exist (Stahl, 2004; Doege & Stah, 2006; Glatz, Luiken & Bonen, 2010; Toprak et al., 2020): plasma membrane fatty acid binding protein (FABPpm), which acts as an integral membrane protein, fatty acid translocase (FAT/CD36), and fatty acid transport proteins (FATPs), a large family of multifunctional proteins. Selected examples of intracellular transporters in insects are presented in Table 4

**Table 4:** Examples of lipophorin receptors in insects.

	**Insect**	**Function of protein**	**Literature data**
**Fatty acid binding protein (FATB)**	*Aedes aegyptii*	function not established yet	Zheng, Blair & Bradley (2013)
	*Aedes albopictus*	lipid storage before diapause	Reynolds et al. (2012)
	*Anopheles gambiae*	function not established yet	Esteves & Ehrlich (2006); Zheng, Blair & Bradley (2013)
	*Apis mellifera*	retinoic acid binding	Evans & Wheeler (1999); Esteves & Ehrlich (2006);Zheng, Blair & Bradley (2013)
	*Culex pipiens*	fat storage during early diapause	Sim & Denlinger (2009)
	*Dipetalogaster maxima*	transport of FFAs in the thoracic muscles during flight	Cavagnari et al. (2000)
	*Drosophila melanogaster*	mediated signalling in both sleep/ wake and memory-related processes	Gerstner et al. (2011)
	*Panstrongylus megistus*	transfer of FFAs to developing oocytes by a non-endocytic mechanism and then segregation into lipid droplets	Fruttero et al. (2011)
	*Schistocerca gregaria*	transport of FFAs to the flight muscle during flight	Wu & Haunerland (2001); Rajapakse et al. (2019)
**Fatty acid translocase (FAT/CD36)**	*Aldrichina grahami*	an important component of protecting olfactory function	Han et al. (2020)
	*Aulacocentrum confusum*	function not established yet	Li et al. (2021)
	*Bombyx mori*	selective carotenoid transport for cocoon coloration; transport of FFAs in the larvae midgut	Sakudoh et al. (2010); Zhang et al. (2020))
	*Calliphora stygia*	function not established yet	Leitch et al. (2015)
	*Cotesia vestalis*	function not established yet	Liu et al. (2020)
	*Drosophila melanogaster*	involved in the oviposition response to C6–C9 fatty acids	Scheuermann & Smith (2019)
	*Galleria mellonella*	function not established yet	Li et al. (2021)
	*Hermetia illucens*	mediates responses to lipid pheromones	Xu et al. (2020)
	*Microplitis mediator*	involved in perceiving plant volatiles and sex pheromones	Shan et al. (2020)
	*Phormia regina*	might solubilize and deliver palmitic, stearic, oleic, and linoleic acids to the midgut during feeding	Ishida, Ishibashi & Leal (2013)
	*Rhodnius prolixus*	function not established yet	Ribeiro et al. (2014)
**Fatty acid transport protein (FATP)**	*Anopheles gambiae*	functionally implicated in cuticular hydrocarbons biosynthesis in oenocytes	Grigoraki et al, (2020a), Grigoraki et al, (2020b)
	*Apis mellifera*	transport of fatty acids for the production of ethyl oleate from the fat body to the honey crop/oesophagus, could be responsible for the transport/removal of ethyl oleate	Castillo et al. (2012)
	*Bombus terrestris*	mediate uptake of extracellular FFAs in the pheromone gland	Buček et al. (2016)
	*Drosophila melanogaster*	transport of FFAs; synthesis of VLCFA; mediating in FFAs uptake into the nervous system	Rittschof & Schirmeier (2018); Van Den Brink et al. (2018); Poidevin et al. (2021)
	*Musca domestica*	FFAs uptake in the midgut	Barroso et al. (2021)
	*Rhodnius prolixus*	function not established yet	Alves-Bezerra et al. (2016)

### Fatty acid binding protein (FABP)

Fatty acid binding protein (FABP) is an evolutionarily-conserved membrane-bound protein that facilitates the uptake of extracellular long chain fatty acids; in adipocytes, localization of FABP into the plasma membrane causes specific and saturable uptake of long chain fatty acids (Schaffer & Lodish, 1994). Some reports clearly demonstrate the presence of FABPs in the midgut epithelium of the investigated insect, hence suggesting a function in dietary lipid uptake (Holtof et al., 2019). Once the FAs pass through the plasma membrane, they are bound by FABPs and transported to different compartments, such as lipid droplets, the endoplasmic reticulum or the mitochondria, depending on the physiological needs of the insect. FABPs might be also involved in trafficking amongst these compartments (Toprak et al., 2020). Examples of FABPs in insects are presented in Table 3

*fabp* expression was upregulated in early diapausing *C. pipiens* females, and the level of expression increased during diapause. In the early stage of diapause, FABP might be involved in mobilizing TAG for storage in the fat body, while in the late stage, FABP may play a role in mobilizing and distributing fatty acids for use in generating energy. The hypothesis that FABP mobilizes fatty acids in late diapause is also supported by the strong upregulation of the putative lipase genes (Sim & Denlinger, 2009; Gondim et al., 2018).

A form of FABP was also purified from the cytosolic fraction of the triatomine *D. maximus* The protein has an apparent molecular mass of 14 kDa, and its N-terminus is unblocked, and the obtained sequence indicates that this FABP belongs to the heart type, characteristic of organisms which obtain energy from fatty acid beta oxidation (Cavagnari et al., 2000).

In adult *Drosophila*, FABP is very strongly expressed in the adult eye, hindgut, fat body, heart (Dourlen et al., 2015). Beyond its role in fatty acid transport, FABP has also been implicated as a modulator of sleep in *D. melanogaster* (Gerstner et al., 2011).

FABP has also been detected in the flight muscle of the migratory locust *Schistocerca gregaria* (Orthoptera: Acrididae) (Haunerland et al., 1992); this insect is not only an economically damaging pest for a wide range of crops but it is also a source of therapeutic sterols (Cheseto et al., 2015).In addition, knockdown of the gene associated with the expression of FABP in *S. gregaria* impairs the ability to fly (Rajapakse et al., 2019).

### Fatty acid translocase (FAT/CD36)

The FAT/CD36 protein was initially identified in the olfactory receptor neurons of Lepidoptera and Diptera. Examples of this proteins in insects are presented in Table 3 It is thought to play a role in odour detection by interacting with extracellular proteins such as odorant binding proteins (OBP) (Xu et al., 2020; Zhang et al., 2020). It has been proposed that *Ae. aegypti* odorant binding protein 22 (AeOBP22) might bind long-chain fatty acids (C15-C20) for example arachidonic acid, and that this process is enhanced by a conformational change in the C-terminal tail that generates a high affinity binding site (Jones, Wang & Murphy, 2019; Wang et al., 2020).

The two OBP *D. melanogaster* proteins expressed in the leg sensilla, *viz.* OBP57d and OBP57e, are involved in the oviposition response to C6–C9 fatty acids. Flies knocked down for either of these two OBPs showed an altered preference for the tested fatty acid compared with control flies (Scheuermann & Smith, 2019).

Although OBP was initially found in the olfactory appendages, these proteins were later discovered in other chemosensory and non-chemosensory organs, such as the midgut (Rihani, Ferveur & Briand, 2021) Fluorescent binding assays revealed that in forensic important blowfly *Phormia regina* (Diptera: Calliphoridae) OBP (PregOBP56a) binds palmitic, stearic, oleic, and linoleic acids, indicating that PregOBP56a might solubilize and deliver fatty acids to the midgut during feeding (Ishida, Ishibashi & Leal, 2013). Similarly, OBP expression has also been detected in the midgut of *R. prolixus* (Ribeiro et al., 2014).

Besides OBPs, another important group of FAT/CD36 are sensory neuron membrane proteins (SNMPs) (Cassau & Krieger, 2020). A transcriptomic study on the antennae of adult *Aldrichina grahami* (Diptera: Calliphoridae), a necrophagous insect with forensic significance, demonstrated differences in the composition and expression of SNMP genes between sex and tissue type. Such differences are likely to greatly influence insect behaviour around a corpse. In addition, a better understanding of the function of candidate olfactory genes may provide a greater insight into the molecular mechanisms of olfactory detection of forensically-important fly species; such information would be of value in when determining the period before minimum post-mortem inference (PMImin) (Han et al., 2020).

In *Drosophila*, SNMP1 was shown to facilitate the detection of lipid-derived pheromones by their cognate receptors in olfactory cilia; structure–activity dissection indicates that the ectodomain of SNMP1 plays an essential role in cilia localization and pheromone-evoked responses (Gomez-Diaz et al., 2016). In addition, both SNMP1 and SNMP2 have been detected in the chemosensory tissues, particularly in the antennae of the hemimetabolous desert locust *S. gregaria* (Jiang et al., 2016). The fact that neuronal-expressed SNMPs and CD36 share analogous functions in insects and vertebrates suggests that the olfactory mechanisms used in the detection of lipophilic odorants may be evolutionarily conserved (Cassau & Krieger, 2020).

In the Lepidoptera, research has shown the presence of three classes of SNMP: SNMP1, which is restrictively expressed in adult antennae, SNMP2, which is broadly expressed in multiple tissues and SNMP3, which is generally concentrated in larval and the adult midgut, indicating a possible function in fatty acid transportation (Zhang et al., 2020). In the model organism *B. mori* (Meng, Zhu & Chen, 2017; Abdelli, Peng & Keping, 2018), it has been proposed that class B type I scavenger receptors belonging to the CD36 protein family might be involved in the active transport of dietary fatty acids across the midgut membrane; this would play a role in elective carotenoid transport for cocoon coloration (Sakudoh et al., 2010).

### Fatty acid transport protein (FATP)

Fatty acid transport protein (FATP) is an evolutionarily-conserved membrane-bound protein that facilitates the uptake of extracellular long chain fatty acid (LCFA) isoforms, which are widely expressed, but with distinct tissue specificity. Their activity is associated with fatty acyl-coenzyme A (CoA) synthetases, which catalyse the formation of CoA-derivatives, an activation step essential for the subsequent metabolic transformation of FAs (Glatz, Luiken & Bonen, 2010). Some research suggests that this protein plays a crucial role in lipid accumulation in insect oocytes (Ziegler & Van Antwerpen, 2006). Examples of FATP in insects are presented in Table 3

The *Drosophila* genome encodes three FATP homologues, Fatp, CG3394 and CG44252. Of these, *Fatp* appears to be expressed in hemolymph–brain barrier (HBB)-forming glial cells, and it shows high homology to mouse and human FATP-1 and FATP-4; as such, this protein is considered as a good candidate for mediating fatty acid uptake into the nervous system (Rittschof & Schirmeier, 2018). In invertebrates, FATP expression is also tissue specific. In adult Drosophila, *fatp* is thought to be the major member of the family as it is very strongly expressed in the adult eye, hindgut, fat body, heart and carcass, whereas *fatp* paralogs, CG3394 and CG30194, are only moderately expressed in some of the tissues of the adult fly (Dourlen et al., 2015).

The two homologs of FATP, *viz.* MdFATP1 and MdFATP2, were found in *M. domestica* (Barroso et al., 2021). In *M. domestica* larvae, FATP1 may enhance the uptake of FAs at the apical membrane of midgut cells, as suggested by its occurrence at midgut microvillar-enriched membrane preparations along the midgut. On the other hand, FATP2, which has a predicted transmembrane domain, but is absent from microvillar-enriched membrane preparations, may be involved in the uptake of FAs through intracellular membranes, such as human FATP4 in the endoplasmic reticulum of enterocytes (Stahl et al., 1999). It is important to note the posterior midgut cells of *M. domestica*, where most of the FATP2 is expressed, have a well-developed endoplasmic reticulum (Terra et al., 1988). (Terra et al., 1988).

The honey bee, *Apis mellifora*, has been the source of a range of medicinal products, such as honey, propolis and bee venom for thousands of years. More recently, it has been used as an important model organism for studying the genetics of behaviour and cognition, as well as the regulation and maintenance of complex societies and their evolution (Oldroyd & Thompson, 2006; Sforcin, Bankova & Kuropatnicki, 2017; Webster, 2019; Al-Hatamleh et al., 2020; Duffy et al., 2020). Studies have also found the expression sites of FATP along the oesophagus to correspond with the intake of nectar by the bee, and the resulting exposure to ethanol. These proteins probably take part in the formation of ethyl oleate, by condensing oleic acid with ethanol and providing free fatty acids, or possibly removing the ethyl oleate from the regurgitate. A search in the honey bee peptide map for these genes resulted in the identification of peptides in the head of the adult worker and 33.6% of these genes were genes coding the FATP (Castillo et al., 2012).

Research suggests that FATP plays an essential role in bombykol biosynthesis in the silkworm *Bombyx mori,* a model animal used in toxicological research and for studying human disease: silkworm genes demonstrate high homology with certain genes related to human hereditary disease (Meng, Zhu & Chen, 2017; Abdelli, Peng & Keping, 2018). FATP stimulates both lipid accumulation and triacylglycerol synthesis via vectorial acylation during pheromonogenesis, thus allowing the uptake of extracellular fatty acids and their subsequent activation to CoA thioesters (Ohnishi et al., 2009).

In addition, a range of transcripts coding FATPs (FATP-1–FATP-3) was identified in the buff-tailed bumblebee, *Bombus terrestris* (Hymenoptera: Apidae), another important evolutionary model organism (Baer, 2003; Goulson, 2004; Wilfert et al., 2007; Rother et al., 2021). These were believed to code for FA transport-related proteins, some of which were homologous to *B. mori* FATPs, which mediate the uptake of extracellular FAs in the pheromone gland (Ohnishi et al., 2009). FATP-1 was abundantly and specifically expressed in the fat body, whereas FATP-2 and FATP-3 were expressed at comparable levels across the tissues, indicating that they play a role in the primary fatty acid metabolism rather than the biosynthesis of fatty acid-derived marking pheromones localized in the labial gland (Buček et al., 2016).

### Metabolism of FFAs

After being absorbed, predigested lipid molecules are used as precursors for the synthesis of more complex structures that will be transported to other organs. Specifically, FFAs are either oxidized in the mitochondria (β-oxidation) for energy production or used to synthesize TAGs, DAGs and phospholipids (Toprak et al., 2020).

Once the FFAs are transported into the cells, they are activated by linking to CoA to generate ‘fatty acyl-CoA (FA-CoA), an intermediate used for FA elongation and β-oxidation (Majerowicz & Gondim, 2013). The acetyl-CoA enters the mitochondrial Krebs cycle to generate energy in the presence of oxygen. β-Oxidation in the mitochondria involves sequential removal of acetyl-CoA and two pairs of hydrogen atoms from the FA-CoA to generate flavin adenine dinucleotide (FADH2) and nicotinamide adenine dinucleotide. Therefore, production of acetyl-CoA is a key intermediate for the interconversion of fats and carbohydrates. When the insect has enough energy, acetyl-CoA is removed from the Krebs cycle and is transported from mitochondria to the cytosol; the acetyl-CoA is then able to enter the citric acid cycle. Notably, acetyl-CoA forms an ester with carnitine before it crosses the inner mitochondrial membrane, which facilitates the transport from mitochondria to the cytosol (Toprak et al., 2020).

Lipolysis is the process based on the constant hydrolysis of TAGs to fatty acids. In this process, the TAGs are first hydrolyzed to DGs and monoglycerides (MGs); these are further transformed into three fatty acid molecules and a glycerol molecule (Skowronek, Wójcik & Strachecka, 2021) All lipolysis is regulated by the protein perilipine, which prevents or stimulates the hydrolysis/phosphorylation of TAG. Its activity increases during starvation and the action of glucocorticoids, and decreases with food consumption. It stimulates phosphorylation and facilitates its translocation from the cytosol of the cell to the surface of the fat droplets, where TG hydrolysis occurs (Skowronek, Wójcik & Strachecka, 2021)

### Biosynthesis of FFAs

**Figure 2:**
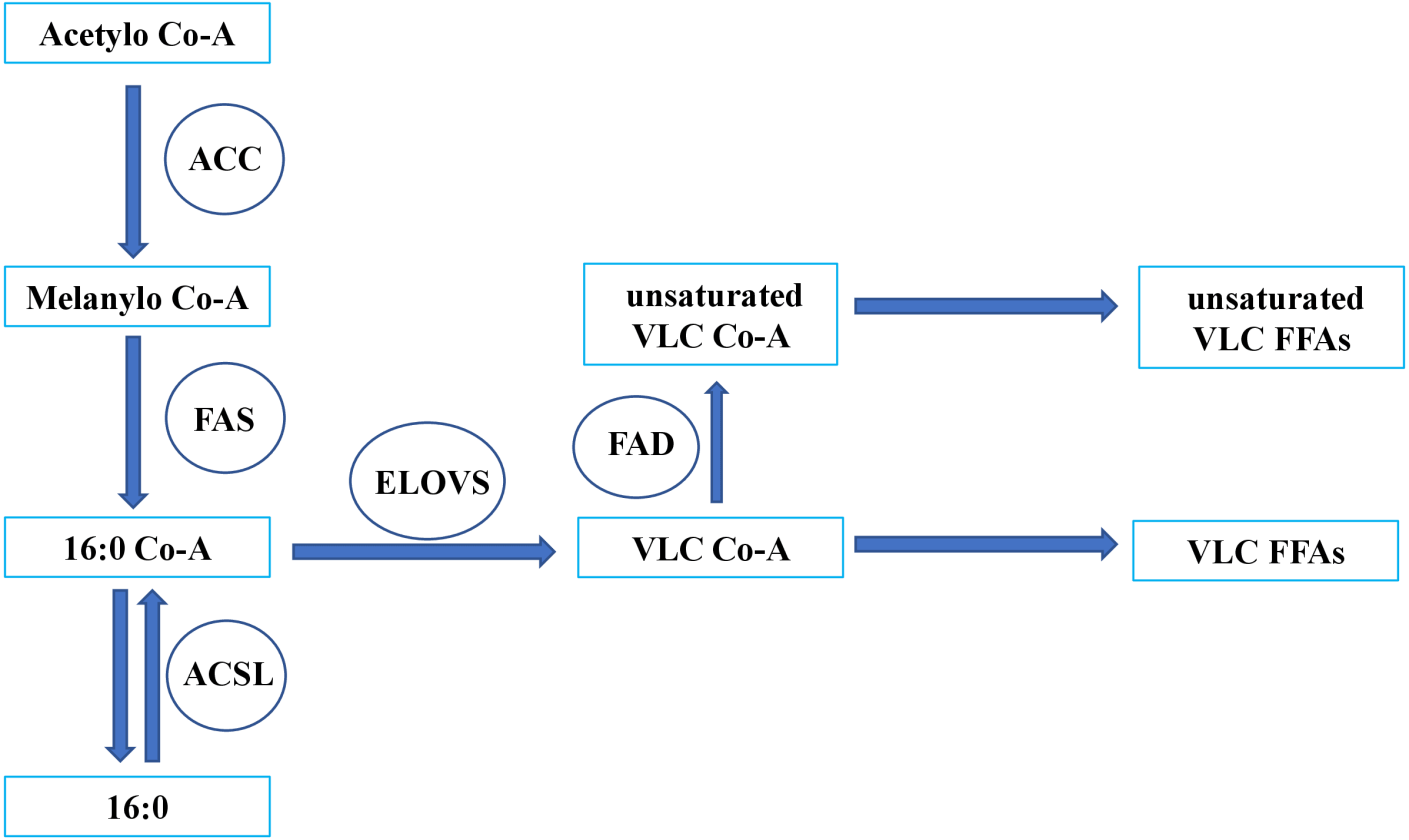
The biosynthesis of FFAs in insect. ACC, acetyl-CoA carboxylase; FAS, fatty acid synthase; ACSL, long-chain acyl-CoA synthetase; FAD, fatty acid desaturase; VLC FFAs, very long chain free fatty.

Although the fatty acid profiles of insects are greatly altered by their diet (Kazek et al., 2019; Kaczmarek et al., 2021), there are some exceptions. The biosynthesis of palmitic, stearic, oleic acid seems to be widespread among insects, and correspondingly these fatty acids are the most abundant in their bodies (De Renobales et al., 1987; Stanley-Samuelson et al., 1988; Gołębiowski et al., 2013a; Malcicka, Visser & Ellers, 2018; Broschwitz et al., 2021). The examples of enzymes engaged in FFAs synthesis in insects are presented in Table 5 and a schema of FFA synthesis is presented in Fig. 2

**Table 5:** Enzymes engaged in FFA metabolism.

**Enzyme**	**Function**		**Insect**	**Literature data**
**Acetyl-CoA carboxylase (ACC)**	catalyze the carboxylation of acetyl-CoA to produce malonyl-CoA	*Aedes aegypti*	Alabaster et al. (2011)
		*Culex pipiens*	Sim & Denlinger (2009)
		*Drosophila melanogaster*	Parvy et al. (2012)
		*Glossina morsitans*	Dean Goldring & Read (1994)
		*Rhodnius prolixus*	Saraiva et al. (2021)
		*Sarcophaga nodosa*	Dean Goldring & Read (1993)
		*Sarcophaga tibialis*	Konji, Olembo & Pearson (1984)
**Fatty acid desaturase (FAD)**	catalyze the removal of hydrogen from an inactivated fatty acyl at a precise position along the hydrocarbon chain	*Aedes aegypti*	Sanders et al. (2003)
			*Aedes albopictus*	Reynolds et al. (2012)
			*Anopheles coluzzii*	Ferdous et al. (2021)
			*Anopheles gambiae*	Helmkampf, Cash & Gadau (2015)
			*Culex pipiens*	Sim & Denlinger (2009)
			*Drosophila melanogaster*	Wicker-Thomas, Henriet & Dallerac (1998); Dallerac et al. (2000)
			*Musca domestica*	Wang et al. (1982); Eigenheer et al. (2002)
			*Nasonia vitripennis*	Brandstetter & Ruther (2016); Semmelmann et al. (2019)
			*Rhodnius prolixus*	Majerowicz et al. (2017)
			*Sarcophaga crassipalpis*	Rinehart, Robich & Denlinger (2010)
**Fatty acid synthase (FAS)**	catalyze the synthesis of palmitate (C16:0, a long-chain saturated fatty acid) from acetyl-CoA and malonyl-CoA,	*Aedes aegypti*	Sanders et al. (2003); Alabaster et al. (2011); Martins et al. (2011)
			*Anopheles gambiae*	Grigoraki et al. (2020b)
			*Blattella germanica*	Juárez, Chase & Blomquist (1992)
			*Culex pipiens*	Sim & Denlinger (2009)
			*Lucilia sericata*	Thompson, Barlow & Douglas (1975)
			*Musca domestica*	Gu et al. (1997)
			*Rhodnius prolixus*	Moriconi et al. (2019)
			*Triatoma infestans*	Juarez, Ayala & Brenner (1996); Juárez & Napolitano (2000); Calderón-Fernández et al. (2017)
**Very long-chain fatty acid elongase (ELOVL)**	perform the initial and rate-controlling condensation reaction in the elongation cycle by successive addition of two-carbon units to very long-chain fatty acids	*Aedes aegypti*	Martins et al. (2011)
			*Aedes albopictus*	Urbanski et al. (2010); Reynolds et al. (2012)
			*Anopheles. gambiae*	Goltsev et al. (2009)
			*Blattella germanica*	Juárez (2004)
			*Drosophila melanogaster*	Chertemps et al. (2005)
			*Periplaneta americana*	Vaz et al. (1988)
			*Triatoma infestans*	Juárez & Brenner (1989); Juarez, Ayala & Brenner (1996); Calderón-Fernández et al. (2017)
**Long-chain acyl-CoA synthetase (ACSL)**	adds Coenzyme A to the long chain (C12-20) fatty acids	*Rhodnius prolixus*	Majerowicz et al. (2017)
		*Drosophila melanogaster*	Zhang, Chen & Wang (2009); Zhang et al. (2011)

Some hymenopteran insects, such as *N. vitripennis*, are able to synthesise palmitic and stearic acid from α-D-glucose (Prager, Bruckmann & Ruther, 2019; Ruther, Prager & Pokorny, 2021) and linoleic acid from oleic acid (Broschwitz et al., 2021). The synthesis of linoleic acid is observed in some insects, for example, some species from the Order Hemiptera (*Myzus cerasi*, *Myzus persicae, Prociphilus fraxini folli*, *Planococcus citri, Bemisia argentifolii*), *Isoptera* (*Zootermopsis angusticollis, Coptotermes formosanus, Reticulitermes flavipes*) *Hymenoptera* (*Nasonia vitripennis*), *Neuroptera* (*Chrysoperla carnea*) and *Coleoptera (Tribolium castaneum)* (Malcicka, Visser & Ellers, 2018).

In female *Ae. aegypti*, sugar feeding before a blood meal resulted in lipid accumulation in the fat body, suggesting that *de novo* lipogenesis was active in these insects (Ziegler & Ibrahim, 2001; Zhou, Pennington & Wells, 2004). In these mosquitoes, amino acids derived from the blood meal were also used for *de novo* lipogenesis, which increased oogenesis efficiency (Zhou et al., 2004).

*De novo* lipogenesis may also occur in tissues other than the fat body. In the hematophagous hemipteran *T. infestans*, fatty acids and hydrocarbons are synthesized from acetate in the integument tissue, resulting in the production of cuticle lipids (Juarez & Brenner, 1989).

### Acetyl-CoA carboxylase (ACC)

The first step in *de novo* fatty acid synthesis is the carboxylation of acetyl-CoA to produce malonyl-CoA, which is catalyzed by acetyl-CoA carboxylase (ACC). Following this, malonyl-CoA units are sequentially condensed with acetyl-CoA to synthesise long-chain fatty acids (Toprak et al., 2020). Phylogenetic analysis has found that, similar to other arthropods but not vertebrates, insects have only one ACC gene (Alabaster et al., 2011; Parvy et al., 2012; Saraiva et al., 2021).

In *R. prolixus* females, the ACC (*RhoprACC*) transcript levels were similar in the anterior and posterior midgut, fat body and ovary on the fourth day after a blood meal, but higher in the flight muscles. In the fat body, gene expression was higher in fasted females and decreased after a blood meal. In the posterior midgut, it increased after feeding, and no variation was observed in the flight muscle. RhoprACC protein content analysis of the fat body revealed a similar profile to gene expression, with higher protein contents observed before feeding and in the first two days after a blood meal. *De novo* lipogenesis was very low in starved females but increased during the initial days after a blood meal. The flight muscles had a very low capacity to synthesize lipids when compared to the fat body (Saraiva et al., 2021).

In *Ae. aegyptii*, ACC transcription in the fat body was stimulated by a blood meal. Research has shown that ACC-deficient mosquito females produced defective oocytes, which lacked an intact eggshell and gave rise to inviable eggs. This severe phenotype was restricted to the 1st gonotrophic cycle, suggesting that the eggshell defect was due to ACC deficiencies in the follicular epithelial cells, which are replaced after each gonotrophic cycle. On the other hand, the knockdown of FAS1 showed delayed blood meal digestion, suggesting that a feedback control mechanism may coordinate the rates of fat body lipid biosynthesis and midgut digestion during feeding. The knockdown of both ACC and FAS resulted in reduced number of laid eggs in both the 1st and 2nd gonotrophic cycles (Alabaster et al., 2011).

In the adult tsetse fly *G. morsitans*, ACC activity increased between two and three days after pupation in the abdomens of male and female flies. In in previously starved flies, a 50–70% increase in ACC specific activity was observed in the thorax, the abdominal cuticle and particularly the fat body within 20 h after a blood meal.

### Fatty acid synthase (FAS)

Insect microsomal and cytosolic FASs were reported in the oenocyte-rich integument of insects. Studies on *B. germanica*, *M. domestica*, and *T. infestans* all confirmed that a microsomal FAS protein capable of adding methylmalonyl-CoA onto methyl-branched fatty acids more effectively than a cytosolic FAS (Juarez, Chase & Blomquist, 1992; Blomquist et al., 1995; Juarez, Ayala & Brenner, 1996)

Three FAS genes have been reported in the *R. prolixus* genome: *RPRC000269*, *RPRC000123* and *RPRC002909* It is worth noting that the fruit fly microsomal FAS (*mFAS)* of the oenocytes clusters is closely related to the *R. prolixus* cytosolic fat body FAS gene and more distantly to the other FAS gene expressed in the oenocytes (Majerowicz et al., 2017). The putative *R. prolixusm* FAS gene (*RPRC002909*) is located immediately downstream from the putative fat body FAS gene (*RPRC000269*) in the supercontig sequence. Similarly, a similar arrangement is observed in *Drosophila*, with the genes *CG3523* (fat body FAS) and *CG3524* (mFAS) lying next to each other. This suggests that the mFAS gene, responsible for the synthesis of methyl-branched fatty acids, might have arisen as result of duplication of the fat body FAS gene, which is an orthologue of the FASN gene of mammals and other groups (Majerowicz et al., 2017).

Tissue-specific expression analysis of *B. germanica* FAS genes revealed that *BgFas1*, *BgFas3*, *BgFas4* and *BgFas7* were highly expressed in the integument, whereas *BgFas2* was dominantly expressed in the fat body (Pei et al., 2019). Research indicated also that RNAi knockdown of BgFas1 caused a dramatic reduction of methyl-branched hydrocarbons, a slight decrease of straight-chain hydrocarbons for both internal and external hydrocarbons and also significant reduction of cuticular FFAs (Pei et al., 2019). Moreover, the BgFas1 mRNA levels were correlated with insect molting cycles, and could be induced by long-term mild dryness treatment. Furthermore, BgFas1 suppression accelerated water loss and led to the early death of cockroaches under desiccation (Pei et al., 2019).

The *C. pipiens* FAS gene, identified as *fas-1, fas-2* and *fas-3*, was upregulated in early diapause: in addition, *fas-1* and *fas-2* are believed to be highly expressed in early diapause but are turned off in late diapause. By contrast, putative *fas-3* remains highly expressed throughout diapause. These results suggest that fat accumulation is induced by the genes involved in fatty acid synthesis. RNA interference (RNAi) analysis revealed that the *fas-1* gene and others (*fas-3* and *fabp*) have important roles in fat storage during early diapause. When expression of these genes is suppressed, female mosquitoes fail to sequester the lipids needed for overwintering (Sim & Denlinger, 2009).

### Very long-chain fatty acid elongase (ELOVL)

Both FAS products and fatty acids derived from the diet are further elongated into very long-chain fatty acids (VLCFA) by successive addition of two carbon units. Ten putative genes of elongases engaged in elongation of fatty acid into VLCFA (ELOVLs) were found in the genome of *R. prolixus* (Majerowicz et al., 2017). These include ELOVL7 family members, which elongate acyl-CoAs ranging in length from 16 to 20 carbons (Naganuma et al., 2011). This clade also includes an ELOVL gene of the mosquito *Ae. albopictus*, which is involved in controlling dehydration resistance in diapause eggs through regulation of the formation of hydrocarbons from VLCFA precursors (Urbanski et al., 2010).

### Fatty acid desaturase (FAD)

FAD belongs to a superfamily of oxygen-dependent membrane di-iron containing enzymes that share common features including a conserved tripartite histidine-rich motif coordinating two iron ions in the active centre. These enzymes catalyze the highly energy-demanding removal of hydrogen from an inactivated fatty acyl at a precise position along the hydrocarbon chain. The process involves a reactive oxo-intermediate formed by the activation of molecular oxygen. The net result of the desaturation reaction is the introduction of a double bond into the fatty acyl chain (and reduction of molecular oxygen to water) (Tupec et al., 2017). The insect FADs exhibit high functional diversity and three types of desaturases are involved in placing double bonds in long chain FAs: acyl-lipid, acyl-ACP and acyl-CoA desaturases (Malcicka, Visser & Ellers, 2018).

Nine putative desaturase genes were identified in *R. prolixus* (Majerowicz et al., 2017). Research indicates that two genes (*RPRC000617* and *RPRC6553*) were orthologous to other insect Δ9-desaturase genes, *Daphnia* and the human Δ9-desaturase gene. These genes encode highly-regulated enzymes that catalyze the formation of a carbon–carbon double bond at the ninth position from the carboxyl end of a saturated fatty acid. These enzymes contribute to the formation of the major monounsaturated components of most insect acylglycerols, which are essential for fat storage (Majerowicz et al., 2017). The Δ11 clade remained as an insect-specific family. Within this clade, six *R. prolixus* genes were grouped into four clusters. Although closely related to the Δ9-desaturases, Δ11 enzymes exhibit a wider substrate range and are involved in the production of sex pheromones in Lepidoptera (Blomquist et al., 2005; Majerowicz et al., 2017). In addition, one *R. prolixus* gene (*RPRC013818*) encodes a putative member of the Δ4-desaturase family, which is a less diverse family (Majerowicz et al., 2017). These enzymes are mostly involved in sphingolipid Δ4 desaturation, a relevant process in cell cycle control during *Drosophila* spermatogenesis (Ternes et al., 2002).

### Long-chain acyl-CoA synthetase (ACSL)

Long-chain acyl-CoA synthetases (ACSL) are believed to mediate in the synthesis of long-chain acyl-CoA esters from fatty acid.

In *R. prolixus*, two genes encoding ACSL isoforms (*RhoprAcsl1* and *RhoprAcsl2*) were detected. *RhoprAcsl1* transcripts increased in posterior midgut on the second day after feeding, and *RhoprAcsl2* was highly transcribed on the tenth day. The knockdown of *RhoprAcsl1* did not result in noticeable phenotypes. However, *RhoprACSL2* deficient insects exhibited a 2.5-fold increase in triacylglycerol content in the fat body, and a 90% decrease in fatty acid β-oxidation. *RhoprAcsl2* knockdown also resulted in a 20% increase in lifespan, delayed digestion, 30% reduced oviposition, and 50% reduction in egg hatching. Laid eggs and hatched nymphs showed remarkable alterations in morphology. The authors propose that *RhoprACSL2* is the main contributor for the formation of the intracellular acyl-CoA pool channeled for β-oxidation in the fat body, and is also required for normal reproduction (Alves-Bezerra et al., 2016).

In *D. melanogaster*, an ACSL isoform (dACSL) is required for normal embryonic segmentation and normal TAG accumulation in larvae (Zhang, Chen & Wang, 2009; Zhang et al., 2011). Both maternal and zygotic *dAcsl* are required for embryonic segmentation (Zhang et al., 2011). The Drosophila dACSL is required in the brain optic lobe for the decapentaplegic (Dpp, a BMP-like molecule, Dpp/BMP) production, for the formation of properly aligned glia and neurons, and for the accurate targeting of retinal axons (Zhang, Chen & Wang, 2009).

### Polyunsaturated fatty acids (PUFAs)

Polyunsaturated fatty acids (PUFAs) are usually associated with biomembranes as phospholipid fatty acids. PUFAs are usually stored in the fat body or in other tissues where they are masked by triacylglycerols (Stanley-Samuelson & Dadd, 1983; Blomquist, Borgeson & Vundla, 1991). They are nutritionally essential for larval mosquitoes of *C. pipiens*, and arachidonic acid is essential for the adult to develop proper wing structure (Dadd & Kleinjan, 1979). The proportion and composition of 20:5, 20:4 and 20:3 vary according to life stage and tissue type (Howard & Stanley-Samuelson, 1996). They are considered to be precursors of leukotrienes, thromboxanes and prostaglandins (Stanley-Samuelson & Loher, 1986; Parnanen & Turunen, 1987; Bell & Sargent, 2003; Van Anholt et al., 2004).

One important group of PUFAs are the metabolites of arachidonic acid, also known as eicosanoids (Li et al., 2021; Zhang et al., 2021); these have a range of functions ranging from stimulating oviposition in crickets, and regulating the function of Malpighian tubules in mosquitoes and ants, to controlling thermoregulation in cicadas (Lord, Anderson & Stanley, 2002); arachidonic acid derivatives are also believed to play crucial roles in mediating cellular and humoral immunity in insects (Stanley & Kim, 2014; Kim & Stanley, 2021). They also mediate nodulation and phagocytosis in the larval wax moth, *G. mellonella* (Mandato et al., 1997) important candidate for replacement mammalian models in immunological studies (Buyukguzel et al., 2007; Smith & Casadevall, 2021), and have been found to control the elongation of haemocytes isolated from tobacco hornworms, *M. sexta* (Miller, 2005), microaggregation reactions to bacterial challenge, this being an important step in nodulation (Miller & Stanley, 2001; Miller & Stanley, 2004; Phelps, Miller & Stanley, 2003), prophenoloxidase release from oenocytoids in the beet armyworm *Spodoptera exigua* (Lepidoptera: Noctuidae) (Shrestha & Kim, 2008), behavioural fever responses to infection in the locust *S. gregaria* (Bundey et al., 2003) and biosynthesis of antibacterial proteins in the silkworm, *B. mori* (Morishima et al., 1997). In *Sarcophaga argyrostoma (Diptera: Sarcophagidae)*, eicosanoids take part in NO and lysozyme biosynthesis after bacterial infection (Mohamed et al., 2018), and are important compounds in the LPS-dependent activation of the IMD pathway in *S. peregrina* (Yajima et al., 2003). Besides these important functions, most insects have relatively low PUFA concentrations; (Stanley & Kim, 2020) attribute this to their high sensitivity to oxygen damage, which might cause their peroxidation and, in turn, cellular damage. It is possible, therefore, that maintaining only a small reservoir of PUFAs may be an evolutionary strategy intended to reduce oxidative stress at the intracellular level (Stanley & Kim, 2020).

### Antimicrobial features of cuticular FFAs

A number of studies based on various organisms have found FFA extracts to demonstrate antibacterial and antifungal effects (Harada et al., 2000; Wille & Kydonieus, 2003; Benkendorff et al., 2005; Liu et al., 2019). The activity of each FFA is influenced by its structure, especially the length of the carbon chain and the presence, number, orientation, and position of double bonds; in addition, a key role in the antibacterial activity of an FFA is played by the presence of a hydroxyl group (Zheng et al., 2005). Unsaturated FFAs are more active than saturated FFAs with the same carbon chain length (Zheng et al., 2005; Desbois et al., 2008) and unsaturated FFAs are more active against Gram-positive bacteria than Gram-negative bacteria (Galbraith et al., 1971). Additionally, FFAs with their double bonds in a *cis* orientation have greater antibacterial activity than FFAs with those in the *trans* orientation (Kabara et al., 1972; Feldlaufer et al., 1993).

In study of the effectiveness of various FFA mixtures against bacterial *(Bacillus cereus* (Bacillales: Bacillaceae), *Bacillus subtilis* (Bacillales: Bacillaceae), *Enterococcus faecalis* (Lactobacillales: Enterococcaceae), *Citrobacter freundii* (Enterobacterales: Enterobacteriaceae), *Pseudomonas aeruginosa* (Pseudomonadales: Pseudomonadaceae) *Pseudomonas fluorescens* (Pseudomonadales: Pseudomonadaceae) and fungal strains: *Paecilomyces lilacinus* (Hypocreales: Ophiocordycipitaceae), *Paecilomyces fumosoroseus* (Hypocreales: Ophiocordycipitaceae), *Lecanicillium lecanii* (Hypocreales: Cordycipitaceae), *M. anisopliae* (Hypocreales: Clavicipitaceae), *Beauveria bassiana [Tve-N39], *B. bassiana* [Dv-1/07]* (Hypocreales: Clavicipitaceae). One mixture containing C14:0, C16:1, C16:0, C18:2, C18:1, and C18:0 fatty acids, one of four taken from *Forcipomyia nigra* (Diptera: Ceratopogonidae), was found to be significantly effective, especially toward *B. cereus* and *E. faecalis*; however, the highest antibacterial activity was demonstrated by C9:0, C10:0 and C16:1. In contrast, C14:0, C16:0, C18:0 and C18:1 demonstrated rather poor antifungal activity and did not inhibit the growth of bacteria (Urbanek et al., 2012; Cerkowniak et al., 2013).

The fatty acid extract of *L. sericata* larvae (LFAs –*Lucilia* fatty acid) exhibited effective antibacterial activity against *Staphylococcus aureus* (Bacillales: Staphylococcaceae) and *Streptococcus pneumoniae* (Lactobacillales: Streptococcaceae) with minimal inhibitory concentrations (MICs) of 125 µg/mL and 100 µg/mL, respectively, with the bacterial wall and membrane being the main targets. Furthermore, the LFAs demonstrated a notable anti-biofilm activity against these two bacteria: the mixture both prevented biofilm formation and eradicated mature biofilms (Liu et al., 2019).

The free fatty acids present in insect lipids are biologically-active compounds. Certain short-chain fatty acids, such as caprylic acid, can block the absorption of phosphorus and thiamine by fungi, thereby preventing the development of mould spores. It has been found that cis-unsaturated fatty acids possess strong inhibitory properties (Barnes & Moore, 1997; Wang et al., 2002). The cuticular FFAs play an important role as a resistance factor of fungal infection by the flies *C. vomitoria, C. vicina, Sarcophaga carnaria* (Diptera: Sarcophagidae) and *L. sericata* (Gołębiowski et al., 2008; Gołębiowski et al., 2012a; Gołębiowski et al., 2013a; Gołębiowski et al., 2013c; Gołębiowski et al., 2014a; Gołębiowski et al., 2014b). Cuticular fatty acids are toxic and fungistatic but also may be stimulatory; for example, palmitoleic acid enhances mycelial growth, but is toxic to conidia of *Erynia variabilis* (Entomophthorales: Entomophthoraceae) (Kerwin, 1984), while the toxic effects of palmitoleic acid can be mitigated by the presence of a sufficient concentration of oleic acid.

The larvae of *C. vicina* are resistant to infection by the entomopathogenic cosmopolitan soil fungus *Conidiobolus coronatus* (Entomophthorales: Ancylistaceae) (Gołębiowski et al., 2008). Histological examination of *C. vicina* larvae exposed to sporulating *C. coronatus* colonies found that conidia were unable to germinate on the fly cuticle, thus suggesting the presence of compounds inhibiting spore germination (Boguś et al., 2007). In fact, the cuticular fatty acid profile of *C. vicina* larvae significantly differs from those of *Dendrolimus pini* (Lepidoptera: Lasiocampidae) and *G. mellonella*, which are highly susceptible to fungal infection. The major difference is the presence of C14:0, C16:1 and C20:0 in the cuticle of *C. vicina*; these three fatty acids are absent from the cuticle of *D. pini* and only present in trace amounts in the *G. mellonella* cuticle (Gołębiowski et al., 2008). The FFAs C14:0, 16:0, 16:1, 18:0, 18:1, 18:2, 18:3, 20:0, and 20:1 demonstrated fungistatic activity against *C. coronatus in vitro*, resulting in reduced sporulation and lower hyphal biomass, a lower ability to infect *G. mellonella* larvae and reduced toxicity of the metabolites released into the culture medium by the fungus (Bogus et al., 2010); these results indicate that these fatty acids play a role in resisting fungal attack.

The effectiveness of the cocktail of protease, chitinase and lipase enzymes produced by *C. coronatus* to degrade the primary cuticular constituents (*viz.* proteins, chitin and lipids) has been found to correlate with the chemical profile of the cuticle. The chemical components with positive correlations may be used by the fungus as nutrients, whereas those with negative correlations might be engaged in insect resistance (Bogus et al., 2017; Wrońska et al., 2018; Kaczmarek et al., 2020a).

## Conclusion

The present review describes the role of FFAs in the physiological processes taking place in insects of medical, veterinary and forensic importance. FFAs play key roles in the reproductive processes of these insects and their resistance to pathogens, and are hence of great interest in development of pest control methods. This is of particular importance for the studied species as they present considerable threats to human and animal health and can incur considerable economic losses.

Insects have gained considerable evolutionary success, and this is arguably due in no small part to their ability to build up an effective pathogen defence system comprising the cuticle and humoral immune system, and cellular defence reactions. Among the defences employed by the insect, FFAs are important factors which determine resistance or susceptibilty to microbial infection; they are employed as cuticle compounds and as precursors of eicosanoids, which also play key roles in the mediation of insect cellular and humoral immunity. FFAs also represent significant energy sources, and supply material for pupae during metamorphosis. In addition, altering the ratio of saturated to unsaturated FFAs allows the insect to adapt to cold conditions: an important consideration in insects living in temperate zones during the winter. The present review also describes the properties of FFA-transport proteins, which also play important roles in all insects.

## Additional Information and Declarations

### Data Availability

The following information was supplied regarding data availability: There was no raw data generated in this work.
